# Centralized and Decentralized Delivery of Transgender Health Care Services: A Systematic Review and a Global Expert Survey in 39 Countries

**DOI:** 10.3389/fendo.2021.717914

**Published:** 2021-09-24

**Authors:** Andreas Koehler, Bernhard Strauss, Peer Briken, Daria Szuecs, Timo O. Nieder

**Affiliations:** ^1^ University Medical Center Hamburg-Eppendorf, Institute for Sex Research, Sexual Medicine and Forensic Psychiatry, Hamburg, Germany; ^2^ University Hospital Jena, Institute of Psychosocial Medicine, Psychotherapy, and Psycho-Oncology, Jena, Germany

**Keywords:** transgender health care, transgender health, access to health care, quality of health care, delivery of health care

## Abstract

**Introduction:**

Transgender health care is delivered in both centralized (by one interdisciplinary institution) and decentralized settings (by different medical institutions spread over several locations). However, the health care delivery setting has not gained attention in research so far. Based on a systematic review and a global expert survey, we aim to investigate its role in transgender health care quality.

**Methods:**

We performed two studies. In 2019, we systematically reviewed the literature published in databases (Cochrane, MEDLINE, EMBASE, Web of Science) from January 2000 to April 2019. Secondly, we conducted a cross-sectional global expert survey. To complete the evidence on the question of (de-)centralized delivery of transgender health care, we performed a grey literature search for additional information than the systematic review and the expert survey revealed. These analyses were conducted in 2020.

**Results:**

Eleven articles met the inclusion criteria of the systematic review. 125 participants from 39 countries took part in the expert survey. With insights from the grey literature search, we found transgender health care in Europe was primarily delivered centralized. In most other countries, both centralized and decentralized delivery structures were present. Comprehensive care with medical standards and individual access to care were central topics associated with the different health care delivery settings.

**Discussion:**

The setting in which transgender health care is delivered differs between countries and health systems and could influence different aspects of transgender health care quality. Consequently, it should gain significant attention in clinical practice and future health care research.

## Introduction

Transgender people’s gender identity does not match their sex assigned at birth. Their gender identities may be located binary within the sexes (female, male) or beyond (e.g., agender). The latter might also be referred to as non-binary or genderqueer genders (1). Health care for transgender people is primarily focusing on gender-affirming medical interventions (e.g., hormone therapy) and associated needs (e.g., mental health care). The current 7^th^ version of the standards of care, published by the World Professional Association for Transgender Health (WPATH), and country-specific guidelines outline the relevance of gender-affirming medical treatment and high-quality care for transgender health (2, 3). The majority of previous research found a positive effect of gender-affirming medical interventions on the health of transgender people (4, 5). A high number of follow-up studies indicate benefits from hormone therapy and gender-affirming surgery (6–8) as well as from other treatment options, e.g., phonosurgery (9). In general, it has been shown that evidence-based gender-affirming medical approaches, in which the transgender person’s gender is respected and safe space to explore their gender is created, improves mental health, quality of life, and several additional factors, e. g., substance abuse (10).

**Figure 1:**
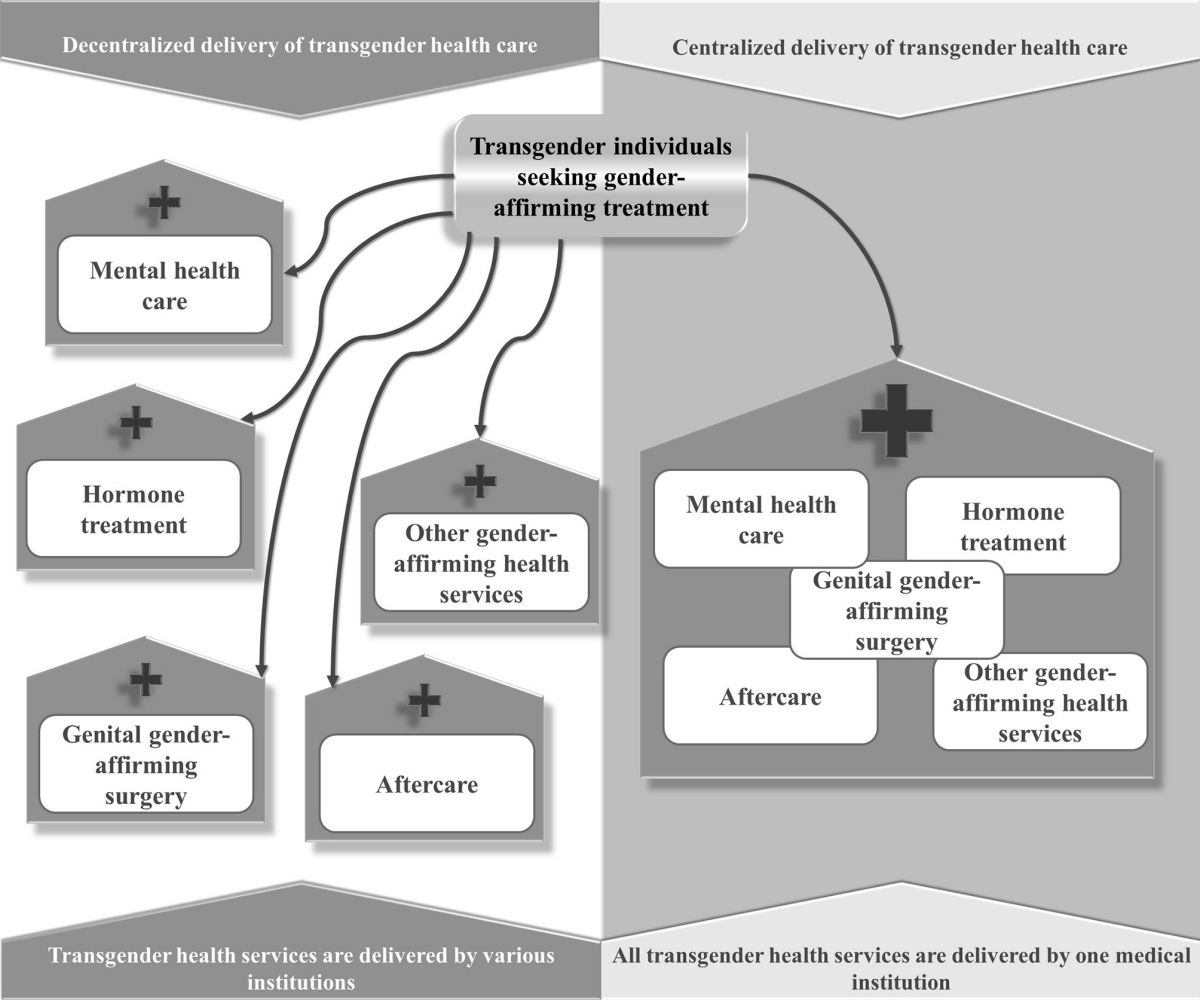
Centralized and decentralized delivery of transgender health care.

To date, transgender health care is delivered in both centralized and decentralized settings (11, 12). A centralized setting of delivery can be described as an interdisciplinary institution that can provide all relevant transition-related treatment options at one location. Decentralized delivery of transgender health care, in contrast, is characterized as the provision of gender-affirming medical treatments by different medical institutions (**Figure 1**). The gender clinic or “gender unit”, a specialized institution especially common at University Medical Centers in Europe (13), often represents a centralized transgender health care service (14, 15). Historically, transgender health service delivery was organized in a centralized setting in the U.S., too. In the 1960s and 1970s, an increasing number of university-based centers offered health care for transgender people (e. g., Johns Hopkins, Stanford University) (16). However, the influence of Paul McHugh, who became the director of the Department of Psychiatry at Johns Hopkins in 1975 (16–18), and a methodologically questionable study (e.g., underpowered sample, inadequate outcome criteria (19) by Meyer and Rether (20), led to the closing of university-based clinics offering gender-affirming medical treatment (16). The study by Meyer and Rether suggested that gender-affirming health care has no health benefit for transgender people [for more details on the history of transgender health care in the U.S see (21)]. This led to the current situation of transgender health service delivery in the U.S., with private practices and community health centers mainly offering primary care for transgender people (e. g., hormone therapy) and university-based departments offering single gender-affirming medical interventions, e. g., gender-affirming surgery (12, 22). However, in both Europe and the U.S., other types of health care delivery exist (i.e., decentralized structures in Europe and centralized structures in the U.S.). In other parts of the world, health care delivery structures are not even developed sufficiently to ensure access to health care for transgender people, e.g., in certain African countries (23).

So far, research on the influence of centralized and decentralized health care delivery was primarily conducted on a superordinate political level and was focusing on health policy aspects, e.g., socioeconomic profits of increasing inter-jurisdictional competition between different medical institutions because of decentralization of health care (24). Decentralized health care delivery in practice has been considered as superior for some medical issues, e.g., HIV (25, 26), as it could ensure easy access to medical care in economically underdeveloped regions. For gender-affirming treatments, the factor of (de-)centralization has not gained sufficient attention so far, even though the setting of health service delivery differs between and within countries and, therefore, could be considered a potential influence on the quality and outcome of transgender health care (13, 27, 28).

There is a need to investigate the gaps in transgender health research as well as to identify where health services might produce inequalities and exclusions (29). Therefore, it appears to be an essential research question if and how the setting of health service delivery affects access to health care, influences the quality of services, and alters the outcome of gender-affirming medical treatment in research and clinical practice. We conducted a systematic literature review, an expert survey, and a grey literature search to shed light on how the setting of health care delivery was addressed in transgender health care research so far.

## Methods

### Systematic Review (1^st^ Study)

The present systematic review is registered on PROSPERO (30). Following the PRISMA guidelines (31), we systematically reviewed studies with a follow-up design evaluating at least one gender-affirming medical treatment (e.g., hormone therapy). At least one psychosocial or somatic outcome (e.g., quality of life) had to be assessed. We completed the final literature search on April 1, 2019. Due to an increase in transgender health-related publications since the early 2000s (32), we restricted the search to publications from the year 2000 up to 2019 and used *Cochrane*, *Medline, EMBASE*, and *Web of Science* databases. The search string was as follows:

[(Gender-nonconform*) OR (Gender-diverse) OR (Gender-Identity-Disorder*) OR (GID) OR (Transsexualism) OR (transsex*) OR (gender-dysphor*) OR (Gender-Incongru*) OR (Transgender*) OR (gender-identity)] AND [(follow-up) OR (longitudinal) OR (prospective)].

**Figure 2:**
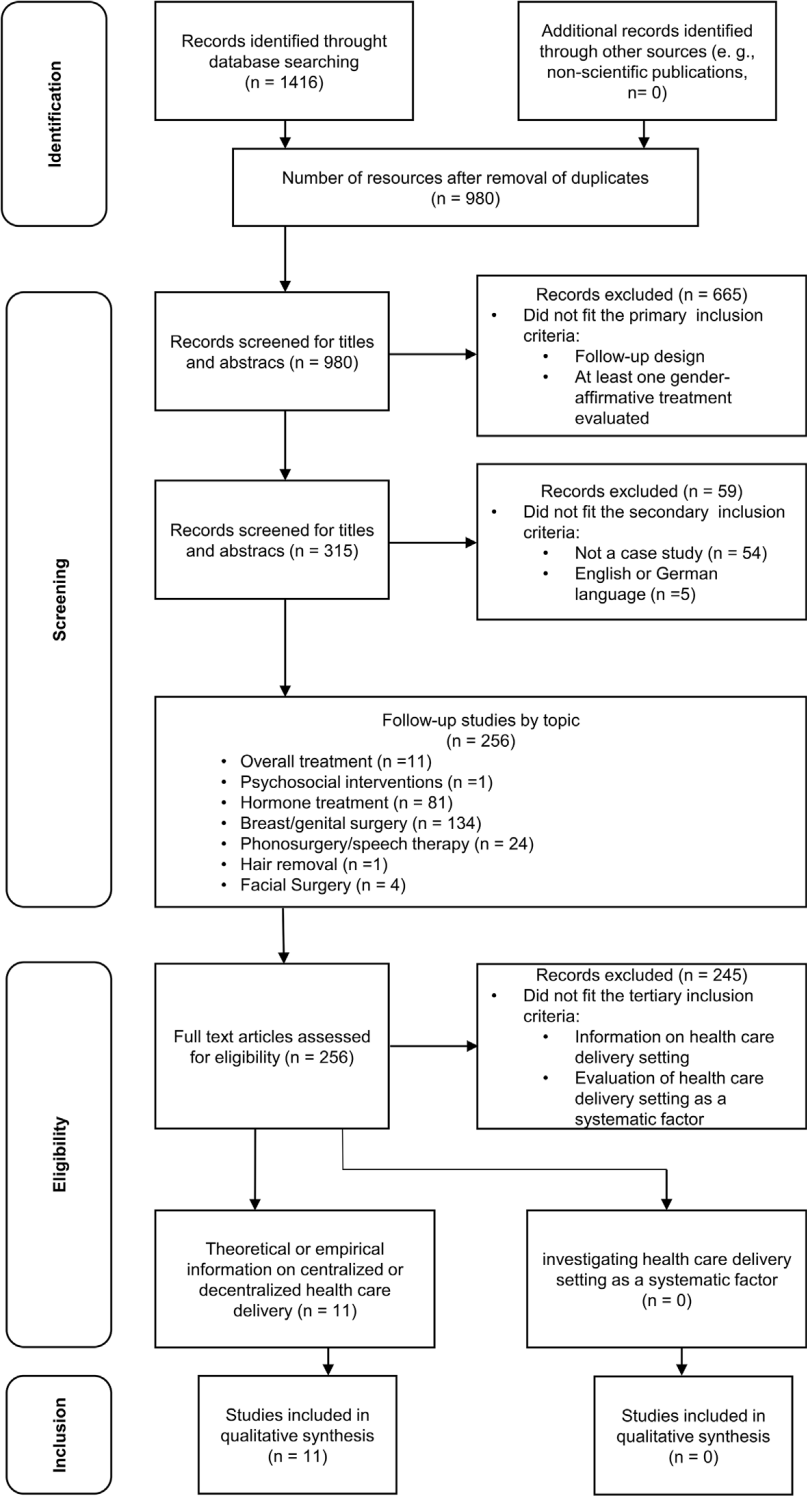
PRISMA Flowchart.

The inclusion criteria were kept as general as possible to ensure high sensitivity to detect potentially relevant studies, as the topic has not gained sufficient attention in research so far. The studies were reviewed in a three-step procedure with primary, secondary, and tertiary inclusion criteria. The primary inclusion criteria included published articles of studies with a follow-up design, evaluating at least one relevant transgender health care service (mental health care, hormone therapy, gender-affirming surgery, speech therapy, hair removal). The secondary inclusion criteria included articles published in English or German language, except for case studies. The tertiary inclusion criteria included published articles with information on the (de-)centralized delivery of transgender health care services (**Figure 2**).

If we were unable to access full texts, the corresponding authors of the article were contacted and asked to provide a copy. The exclusion criteria excluded studies with no follow-up; no evaluation of at least one transgender health care intervention; studies that were limited to qualitative outcomes; and studies that were found in non-scientific publications. We imported eligible articles into Endnote software (Thomson Reuters, Endnote X9, 2018). The first author AK conducted the literature analysis after joint planning with the last author TN, sorting materials based on the primary inclusion criteria after reviewing both title and abstract (“Yes”, “no”, “maybe”). Afterward, the included publications were sorted based on the secondary inclusion criteria after reviewing both title and abstract (“Yes”, “no”, “maybe”). In a third step, the included publications were sorted based on the tertiary inclusion criteria after reviewing the available full texts (“Yes”, “no”, “maybe”). See **Figure 2** for a flowchart of the different steps of the study selection process and results.

The databases from the different selection steps were sorted based on the evaluated transgender health care service (mental health counseling, hormone therapy, gender-affirming surgery, speech therapy, hair removal) and analyzed further (Tables of the databases after applying the first and second selection criteria are available from the corresponding author upon request). After applying the tertiary selection criteria, the final sample included articles of follow-up studies that were further analyzed, focusing on information concerning (de-)centralized delivery of health care services. Extracted data from the articles included authors, study design, country, participants, evaluated gender-affirming medical treatment, outcomes, and information regarding (de-)centralized delivery of health care services.

We used the NIH Quality Assessment Tool for Before-After (Pre-Post) Studies With No Control Group (33) to assess study quality for non-randomized studies. The tool primarily focuses on the internal validity of a study. It, therefore, helps to determine to what extent an outcome can be attributed to an intervention and not to biases or other confounding factors. Amongst others, aspects like representativeness of study participants for the clinical population, description of the intervention(s), or outcome measures were assessed. AK and TN conducted the quality rating of the studies included.

### Expert Survey (2^nd^ Study)

The TransCareExpert survey was a web-based survey designed to investigate the experiences of transgender health care providers and researchers with the centralized and decentralized delivery of transgender health care. Data collection took part between November 2019 and April 2020. This cross-sectional survey was developed in the English language, followed strict ethical guidelines, and received ethical approval from the Local Psychological Ethics Committee (LPEK) at the Center for Psychosocial Medicine, University Medical Center Hamburg-Eppendorf (No.: LPEK-0071, date: 09/15/2019). Informed consent was obtained from all individual participants included in the study.

The survey was open to anyone who worked as a researcher or provider of transgender health care and was at least 16 years of age. Participants were recruited *via* mailing lists of specialized medical associations, the professional network of the authors, and through snowball sampling. We were not able to provide a paper-pencil version of the survey due to the global range of the study and a lack of financial and human resources. Therefore, access to a web-enabled device and technical affinity need to be considered potential biases for participation in the study. By encouraging participants to promote the survey within their professional network, we tried to address these issues.

All questions of the survey were developed by the authors of this article. A pilot study was conducted with ten researchers from different countries who had experience in the field of transgender health care. The survey was reviewed according to the suggestions from the pilot study. The TransCareExpert survey collected demographical data regarding age, gender, and country of practice. Questions regarding professional experiences with providing or researching transgender health care were focusing on the participant’s occupation within transgender health care (e.g., clinician), their (medical) specialty, transgender-specific education (e.g., fellowship training), type of institution the participant works at (e.g., university hospital), and transgender health care services this institution offers. Experiences with working in centralized or decentralized transgender health care delivery settings and a description of the transgender health service system of the country of practice were assessed. Participants were asked to evaluate potential effects of the setting of health care delivery on the involvement of the transgender community into health care provision, the professional exchange between providers, the collaboration with non-medical community-linked institutions, and the collaboration with health insurances. Finally, pros and cons of centralized and decentralized transgender health service delivery were assessed. All aspects were investigated using closed-ended questions with predefined response options, and open-ended, free-response questions (see **Table S9** for the questionnaire).

### Grey Literature Search

We conducted a grey literature search to find further information about the health care delivery setting of the countries included in our study that were not published in peer-reviewed journals or derived from the answers of the expert survey. Therefore, we searched for websites of government agencies, professional organizations, and other organizations. Furthermore, we carried out a grey literature database search using the following databases: OpenGrey, New York Academy of Medicine´s Grey Literature Report. Finally, a general search engine search was conducted using Google web search. AK and DS independently reviewed the identified grey literature compared to the evidence from the systematic review and the expert survey.

### Statistical Analyses

To assess changes in study quality of studies included in the systematic review over the time of publication, linear regression was conducted.

Continuous data of the expert survey are presented as mean (SD). Categorical data are presented as n (%). Chi-square tests were calculated to assess frequency differences between the groups regarding questions on involvement of the community in care, exchange between providers, and collaboration with non-medical institutions and insurances. Free text responses were analyzed following qualitative content analysis (34), where subcategories and superordinated categories are built based on the qualitative material.

The health care delivery setting of a certain country was classified depending on the presence of at least one specialized center offering the most common treatments of transgender health care (e.g., mental health counselling, hormone therapy, genital gender-affirming surgery). Countries without a center were classified as having a decentralized structure of health care delivery. Countries with at least one center, but other non-interdisciplinary health care institutions, were classified as having both a centralized and decentralized structure of transgender health care delivery. Countries where transgender health services are mainly delivered by specialized centers were classified as having a centralized structure of health care delivery. We are aware that this classification does not adequately represent the whole health service delivery system of a certain country, as it appears that in almost every country, transgender health care is also provided by providers in private practice. However, our classification intents to point out, what institutions supply a major part of transgender health service in that certain country.

## Results

**Figure 3:**
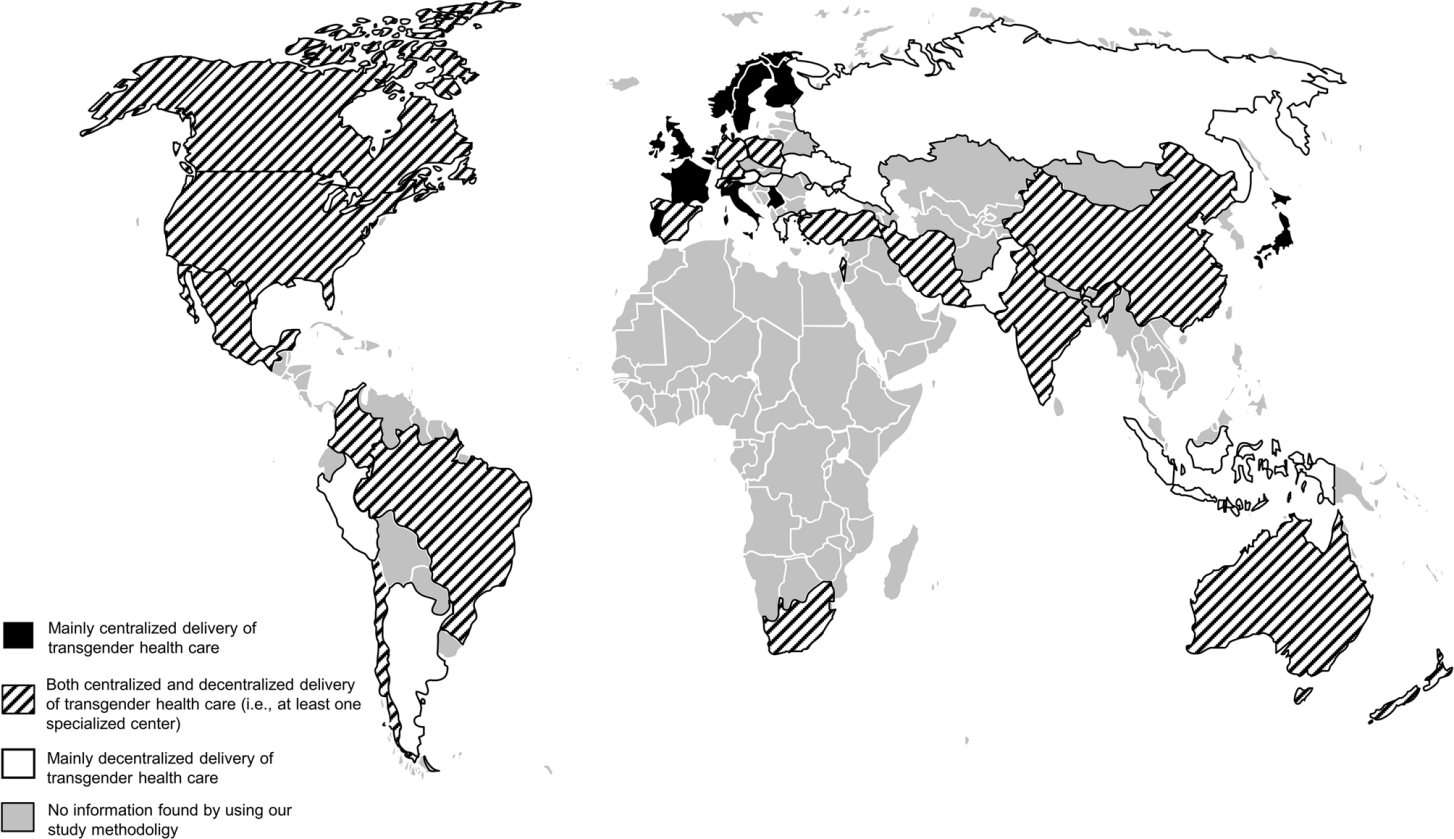
Transgender health care delivery worldwide.

**Figure 4:**
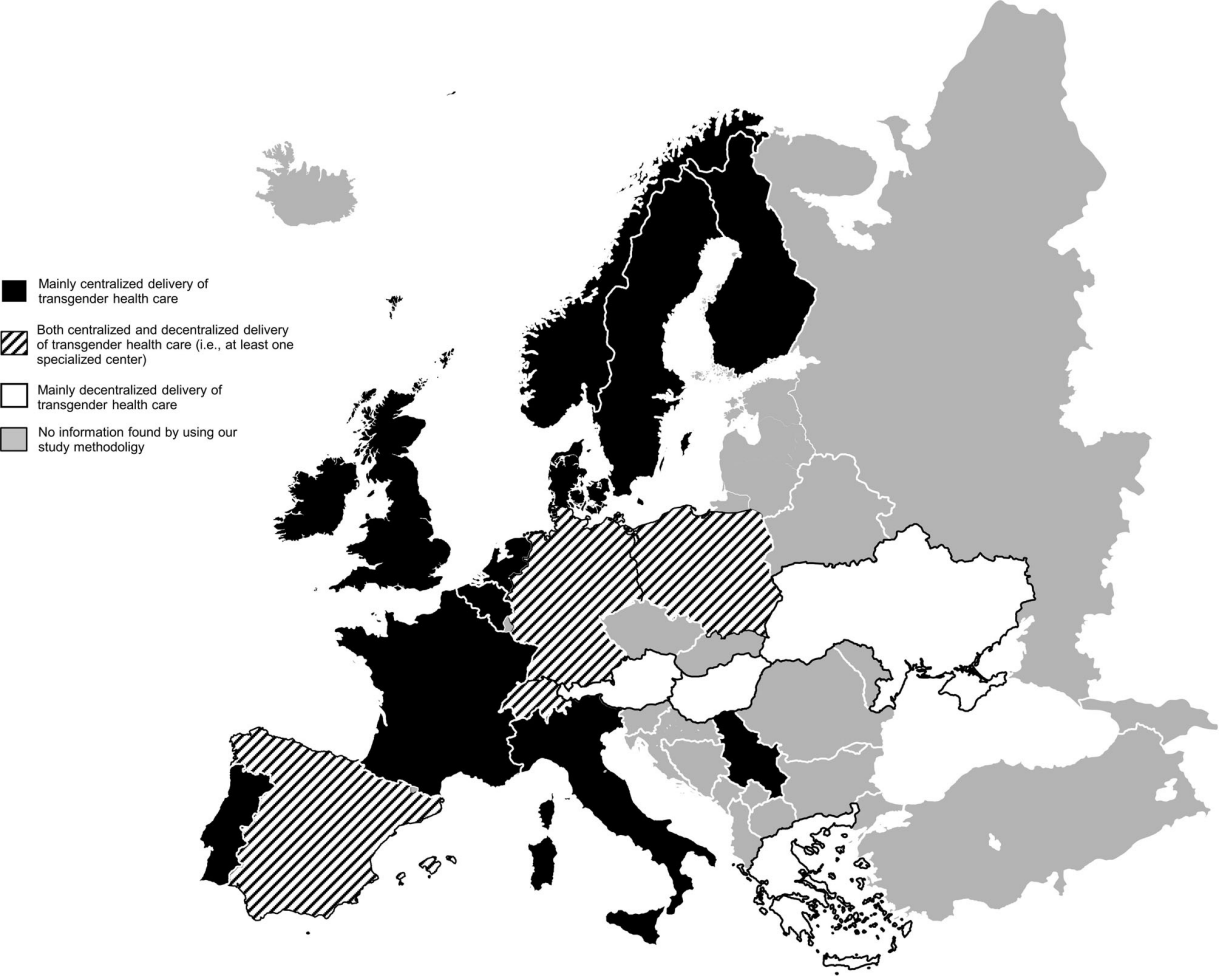
Transgender health care delivery in Europe.

**Figures 3** and **4** summarizes the results from the systematic review, the expert survey, and the grey literature search concerning the structure and (de-)centralization of transgender health service delivery in 40 countries all over the world. Information on 39 countries derived from the systematic review, the expert survey, and the grey literature search. Information on 1 country (Denmark) derived only from the systematic review and the grey literature search. **Table S1** gives a detailed overview over the structures on the national level.

### Systematic Review (1^st^ Study)

We identified a total of 11 articles that fit the primary, secondary, and tertiary inclusion criteria (**Figure 2** and **Table 1**). None of the articles investigated the delivery of health care as a standardized factor (35, 40, 41, 47–54). Hence, the studies included in the qualitative synthesis are providing unsystematic information on the health care delivery structure concerning the extent of centralization.

**Table 1:** Studies included in the systematic review.

Authors	Study design	Country	Participants	Treatment(s) evaluated	Outcome	Information regarding (de-) centralized delivery of health care	Investigating health care delivery setting as a systematic factor
(35)	Single-center, clinical follow-up	Belgium	57 transgender adults	Hormone therapy, gender-affirming surgery	Mental health	- Section of the paper: introduction	No
						- One specialized clinic for the whole country	
						- for insurance coverage, transgender people have to receive treatment at this clinic	
						- the only possibility to bypass the public health care system is undergoing gender-affirming surgery in private sector or abroad	
						- Participants of the study received all procedures from the one specialized clinic	
(36)	single-center, clinical follow-up	Denmark	158 transgender adults	Gender-affirming surgery	Gender distribution, age trends, surgeries performed	- Section of the paper: methods	No
						- All participants of the study received mental health counseling and hormone therapy from the same specialized gender identity service	
(37)	single-center, clinical follow-up	England	201 transgender adolescents	Psychological support, GnRHa treatment	Gender Dysphoria, psychosocial functioning	- Section of the paper: methods	No
						- Participants of the study received all treatments in an interdisciplinary gender clinic	
(38)	Single-center, clinical follow-up	Belgium	55 transgender adults	Gender-affirming surgery	General health, sexual health	- Section of the paper: introduction	No
						- Transgender health care in Europe mostly centralized in nationally sanctioned gender centers	
						- Transgender health care in the U.S. often in a patchwork of community clinics (decentralized)	
(39)	Single-center (community health center), clinical follow-up	United States	57 transgender adults	Hormone therapy	Body mass index, blood pressure, lipids, sex hormone levels, persistence if menses	- Section of the paper: methods	No
						- Six specialized clinics for the whole country offering all treatments necessary for gender affirmation	
						- Participants of the study received treatment in one of the six specialized clinics	
(40)	Population-based matched cohort study	Sweden	324 transgender adults	Gender-affirming surgery	Mortality, psychiatric morbidity	- Section of the paper: methods	No
						- One specialized clinic for the whole country offering all treatments necessary for gender affirmation	
						- Participants of the study received hormone therapy and gender-affirming surgery from the same clinic	
(41)	Single-center, clinical follow-up	The Netherlands	2307 transgender adults	Hormone therapy, gender-affirming surgery	Prostate cancer	- Section of the paper: introduction	No
						- One specialized clinic for the whole country offering all treatments necessary for gender affirmation	
						- Participants of the study received hormone therapy and gender-affirming surgery from the same clinic	
(42)	Multi-center, clinical follow-up	Sweden	42 transgender adults	Gender-affirming surgery	Gender dysphoria, satisfaction with surgery, social functioning, work, relationships, sexuality	- Section of the paper: introduction	No
						- Six specialized clinics for the whole country, two offering gender-affirming surgery	
						- Participants of the study received transgender health care from two of the six specialized clinics	
(43)	Nation-wide cohort study, 30-year period follow-up	Denmark	104 transgender adults	Gender-affirming surgery	Psychiatric morbidity & mortality	- Section of the paper: introduction	No
						- One specialized clinic for the whole country	
						- for insurance coverage, transgender people have to receive treatment at this clinic	
						- Participants of the study received all procedures from the one specialized clinic	
(44)	Nation-wide cohort study, 30-year period follow-up	Denmark	104 transgender adults	Gender-affirming surgery	Somatic morbidity, cause of death	- Section of the paper: introduction	No
						- One specialized clinic for the whole country	
						- for insurance coverage, transgender people have to receive treatment at this clinic	
						- Participants of the study received all treatments from the one specialized clinic	
(45)	Single-center, clinical follow-up	The Netherlands	1254 transgender adults	Hormone therapy	Bone mineral density	- Section of the paper: introduction	No
						- Refers to (46): 95% of the transgender population receives treatment in one specialized clinic offering all treatments necessary for gender affirmation	

None of the studies were randomized controlled trials; seven were single-center clinical follow-up studies (35–39, 41, 45); one was a multi-center clinical follow-up study (42); one was a population-based matched cohort study (40); two were nation-wide cohort studies (43, 44). According to the quality assessment using the NIH Quality Assessment Tool for Before-After (Pre-Post) Studies With No Control Group (33), studies were mostly ranked poor or fair (see supplementary material for the detailed assessment). Linear regression did not show a change in study quality over the time of publication (B=.03, *P*=.45, f²=0.01). The majority of studies were conducted in Europe (n=10) (35–38, 40–45), one was located in the United States (39). Sample sizes ranged from n=42 (42) to n=2307 (41). The average sample size was n=424. Most studies evaluated gender-affirming surgery (n=6) (36, 38, 40, 42–44). N=2 studies investigated general hormone therapy (39, 45), n=2 studies investigated general hormone therapy and gender-affirming surgery (35, 41), and n=1 study evaluated psychological support and specific hormone therapy (GnRHA treatment) (37). N=6 studies reported psychosocial outcomes or sample descriptions only (e.g., mental health, age) (35–38, 40, 42, 43). N=4 studies reported somatic outcomes only (e.g., body mass index) (39, 41, 44, 45).

None of the selected articles investigated the setting of health care service delivery as a systematic factor. N=7 studies reported information about the setting of health care delivery in the introduction (35, 36, 39, 42–45). N=4 studies reported information concerning the setting of health care delivery in the methods section (37, 38, 40, 41). Information regarding the (de-)centralized delivery of health care was given on a systemic and an individual level. Information on a systemic level refers to the superordinated structure of the health care system regarding the delivery of health care in a particular country overall. On an individual level, the study authors gave information regarding the specific delivery structure in which the study was conducted.

N=9 studies reported on a systemic level about centralized health care in the health care system of the country in which the study was conducted (35, 36, 39–45). Six of these studies reported of only one specialized center for the whole country (35, 36, 41, 43–45). The studies were conducted in Denmark (36, 43, 44), the Netherlands (41, 45), and Belgium (35). Two studies gave information on the centralized delivery of transgender health care in Sweden by six hospitals (40, 42). One study (39) addressed differences between the U.S. and Europe regarding the health care delivery setting: transgender health care in Europe is mostly delivered in centralized settings by specialized centers, whereas in the U.S., treatment is provided by a patchwork of community clinics. N=2 studies gave information regarding a centralized setting of transgender health care delivery on an individual level (37, 38). Both studies were conducted in a specialized center. One study was located in England (37), the other in Belgium (38). For Belgium, another study (45) gave information that the clinic the study was conducted in is the only in the country.

### Expert Survey (2^nd^ Study)

125 participants from 39 countries took part in the survey (**Figures 3** and **4**, all marked countries except for Denmark). 57 (45.6%) were involved in medical care for transgender people, 55 (44.0%) were involved in mental health care for transgender people, and 65 (52.0) were researchers. Most of the participants (57, 45.6%) were physicians. On average, participants were 46.4 (SD 11.0) years of age and had 12.5 (SD 8.0) years of experience in working with transgender people. 13 (10.4%) were transgender. 47 (37.6%) worked in a centralized setting of transgender health care delivery, 78 (62.4%) worked in a decentralized setting. Detailed participants’ characteristics are presented in in **Table 2**. Their evaluation of the effects of the health care delivery setting on involvement of the community in care, exchange between providers, and collaboration with non-medical institutions and insurances are presented in **Table S2**. Categories of the pro and contra free text responses regarding centralized and decentralized delivery of transgender health care are summarized in **Table 3** (see **Table S4–S7** for a detailed summary of the answers).

**Table 2:** Participants characteristics from the expert survey.

	N° (%)/Mean (SD)
Total number of participants	125
Involvement in transgender health care	
Medical care	57 (45.6)
Mental health care	55 (44.0)
Research	65 (52.0)
Type of clinician	
Physician	57 (45.6)
(Advanced Practice) Nurse	5 (4.0)
Physician Assistant	4 (3.2)
Psychologist	23 (18.4)
Licensed Clinical Social Worker	5 (4.0)
Other	13 (10.4)
Medical speciality	
Surgery	25 (20.0)
Endocrinology	12 (9.6)
Mental health	51 (40.8)
Other	18 (14.4)
sub-speciality, fellowship training, or licensure comments	62 (49.6)
Years of experience in working with transgender people	12.5 (8.0)
Type of health care institution	
University hospital	47 (37.6)
Non-university hospital	12 (9.6)
Community health center	8 (6.4)
Private practice	42 (33.6)
Other	11 (8.8)
Field of research	
Mental health	36 (28.8)
Children & Adolescents	12 (9.6)
Endocrinology	10 (8.0)
Social Sciences	13 (10.4)
Voice and Communication	1 (0.8)
Surgery	9 (7.2)
Law	5 (4.0)
Other	20 (16.0)
Years of experience in researching transgender health care	10.1 (8.0)
Type of research institution	
University/University hospital	55 (44.0)
Public research institute (not related to a university)	1 (0.8)
Private research institute (not related to a university)	4 (3.2)
Other	5 (4.0)
Age	46.4 (11.0)
Gender	
Man/Male (men of transgender experience and cisgender men)	59 (47.2)
Woman/Female (women of transgender experience and cisgender women)	57 (45.6)
Non-binary/Genderqueer	6 (4.8)
Other	3 (2.4)
Identification as transgender	13 (10.4)
Health care delivery setting participant works in	
centralized	47 (37.6)
decentralized	78 (62.4)
Health care delivery setting of the country participant works in	
(mostly) centralized	31 (24.8)
(mostly) decentralized	41 (32.8)
both	53 (42.4)

**Table 3:** Pros and Cons of (de-)centralized transgender health care delivery.

Centralized health care delivery		Decentralized health care delivery	
Pro	Con	Pro	Con
Comprehensive, interdisciplinary care & professional exchange	Access barriers to health care (travel & waiting list)	Easy access to health care	Lack of expertise
Professional expertise & standardized care	Monopolization of care	Opportunity to choose health care provider & treatment	Fragmentation of care & coordination challenges
Patient-centered care	Detachment from the community	Community-involvement	
Research opportunities	Structural challenges		

### Grey Literature Search

Results from the grey literature search are presented in **Tables S1**, **S8**. Grey literature sources confirmed the information derived from the systematic review and the expert survey and added valuable details to get a comprehensive picture of the transgender health care delivery in the different countries.

## Discussion

As the first of its kind, the present study investigated centralized and decentralized health care delivery structures for transgender health services. With a systematic review of the existing literature, a global expert survey, and a grey literature search, we were able to shed light on this important, but so far unnoticed, aspect of transgender health care and gained valuable knowledge to further improve transgender health care quality.

We found that previous literature was mainly focusing on the centralized delivery of health care in specialized centers, even though none of the included studies investigated the setting of health care service delivery systematically. In various European countries, transgender health care services are delivered by one specialized center [e.g., Belgium (35, 38)] or by a small network of specialized centers [e.g., Sweden (40, 42)]. With further empirical evidence from our expert survey as well as the grey literature search, we conclude that the (university-based) gender-unit is the standard model for transgender health care delivery in many European countries (**Figures 3** and **4**). Interdisciplinary cooperation from various medical departments offers a broad range of gender-affirming medical treatments, often centrally coordinated by a single superordinated department (13, 55). Outside Europe, transgender health care was delivered centralized only in Japan. One study gave information about decentralized delivery of transgender health care in the U.S (39). In the U.S., transgender health care is typically delivered by private providers or in community health centers, where general health and hormone therapy for transgender people is provided (39). Surgical procedures are usually conducted by specialized departments focusing on gender-affirming surgery. However, specialized centers, offering transgender health services in a centralized setting, are also present in the U.S (56, 57). In fact, the presence of both centralized and decentralized institutions in a certain country was the most common structure of transgender health care delivery in our study (**Figures 3** and **4**). In most countries, our study revealed the presence of at least one center specialized in transgender health care offering the most common interventions. However, often only single or a few specialized centers existed, and transgender health care was delivered by a variety of institutions. In a small number of countries (e.g., Russia, Ukraine, Indonesia), transgender health care was delivered exclusively decentralized, without the presence of specialized, interdisciplinary centers.

If and how the health care delivery setting affects the quality of transgender health care was not investigated in prior research so far. By asking on the involvement and active contribution of the transgender community in care, the professional exchange between caregivers, and the collaboration with non-medical community organizations and health insurance, our expert survey did not find a clear pattern which setting of health care delivery could be in favor. Up to 34.0% of the participants of our expert survey reported, that they cannot judge or do not know about these topics, and we found no significant frequency differences favoring on health care setting regarding one of these issues (**Table S2**). As the health care delivery setting has not gained any attention in research so far, providers and researchers might not have a clear position on its effects on their daily practice and were therefore unable to assess positive or negative effects. Therefore, research on a more individual level, e.g., interview studies or focus groups, might be an option to approach this topic in future research with health care providers. Regarding aspects in favor of centralized transgender health care delivery in general, participants of the expert survey mainly focused on the comprehensiveness of care, professional expertise, patient-centeredness, and research. In line with that, several interdisciplinary gender clinics have implemented well-established procedures of transgender health care service delivery that promoted positive outcomes following gender-affirming interventions (13, 57–59). On the other hand, centralized care was negatively brought in connection with access barriers to care, especially due to long travel and waiting lists, the monopolization of care, a detachment from the transgender community, and structural challenges. Pros of decentralized transgender health care delivery were arguments to the contrary, focusing on easy access to care, free choice of providers and treatments, and a better involvement of caregivers into the transgender community. Prior research found the lack of providers with sufficient knowledge is the biggest barrier to health care for transgender people (60). A decentralized delivery structure could face this problem by ensuring better access to transgender health services. However, these health care providers still would need specialized training in gender-affirming care to deliver sufficient transgender health care. Newly developed online training, e.g., by the Global Education Institute of WPATH, could be a first step to make it possible to get high-quality training in gender-affirming care remotely. This could be especially important for providers from rural areas with decentralized structures of health care deliveries and health care systems with lacking financial resources for travel to get in-person training. Moreover, community involvement and partnerships across various stakeholders could maximize the quality of transgender health care and improve health-related outcomes, as it takes issues into account, that are not directly related to the delivery of medical services for transgender people (e.g., employment discrimination) (61). However, cons regarding decentralized care focused on the lack of expertise of health care providers and a fragmentation of care. In sum, comprehensive care with certain medical standards on the one hand, and individual care on the other, were the two central topics of our participants regarding pros and cons of centralized and decentralized delivery of transgender health care. This is in line with prior research, theorizing models of high-quality transgender health care (62). For high-quality, patient-centered transgender health care, however, these two main aspects, and their subcategories (**Table 3**), should not be understood as mutually exclusive for one setting of health service delivery, but rather be integrated and receive equal attention by providers and researchers (63).

The central limitation of the present systematic review is that the (de-)centralized delivery of health care was not systematically assessed as an outcome criterion in the included studies. The review, therefore, confirms that investigating the influence of the health care delivery setting is a so far under-studied perspective in transgender health and documents the need for further research. Moreover, countries from certain regions of the world, e.g., Africa, are underrepresented in the present study. Participants of the expert survey needed access to a web-enabled device and technical affinity, which could have excluded certain providers or researchers, e.g., from developing countries. Finally, a grey literature search is prone to overlook certain evidence.

It has been shown that the setting in which transgender health care is delivered differs between countries and health care systems. Moreover, both delivery settings were assessed as having certain advantages and disadvantages against the other. Taking these issues into account in future research and provision of health services might be a new important component to ensure high-quality transgender health care. E.g., if and how the health care delivery setting could affect the clinical outcome of transition-related health care (e.g., quality of life) is already part of ongoing studies (64). It should get more attention in future research studies and be considered as a potentially important variable that influences the health outcome and the quality of health care.

## Data Availability Statement

We will consider sharing de-identified, individual participant-level data that underlie the results reported in this Article on receipt of a request detailing the study hypothesis and statistical analysis plan. All requests should be sent to the corresponding author. The corresponding author and lead investigators of this study will discuss all requests and make decisions about whether data sharing is appropriate based on the scientific rigour of the proposal. All applicants will be asked to sign a data access agreement.

## Ethics Statement

The studies involving human participants were reviewed and approved by Local Psychological Ethics Committee (LPEK) at the Center for Psychosocial Medicine, University Medical Center Hamburg-Eppendorf (No.: LPEK-0071, date: 09/15/2019). The participants provided their written informed consent to participate in this study.

## Author Contributions

AK contributed to the design of the study, undertook the statistical analysis, managed the literature searches, contributed to the qualitative analysis, wrote the first draft of the manuscript, and contributed to the correction of the manuscript. BS contributed to the design of the study and the correction of the manuscript. PB contributed to the design of the study and the correction of the manuscript. DS managed the literature searches, contributed to the qualitative analysis, and contributed to the correction of the manuscript. TN contributed to the design of the study, managed the literature searches, and contributed to the correction of the manuscript. All authors contributed to the article and approved the submitted version.

## Funding

AK is funded by the Claussen Simon foundation Hamburg, Germany, as a PhD student. There was no further funding for this work.

## Conflict of Interest

AK is funded by the Claussen Simon foundation Hamburg, Germany, as a PhD student.

The remaining authors declare that the research was conducted in the absence of any commercial or financial relationships that could be construed as a potential conflict of interest.

## Publisher’s Note

All claims expressed in this article are solely those of the authors and do not necessarily represent those of their affiliated organizations, or those of the publisher, the editors and the reviewers. Any product that may be evaluated in this article, or claim that may be made by its manufacturer, is not guaranteed or endorsed by the publisher.
