# Supplementary material for: Centralized and Decentralized Delivery of Transgender Health Care Services: A Systematic Review and a Global Expert Survey in 39 Countries
Source: Front Endocrinol (Lausanne). 2021 Sep 24;12:717914. doi: 10.3389/fendo.2021.717914 (PMC8497761; doi:10.3389/fendo.2021.717914)
Supplement: Supplementary file 1 [file Table_1.docx]

Table S1 Health care delivery settings in 40 countries

|  | **Systematic review** | **Expert survey** | | | | **Additional evidence from grey literature search** |
| --- | --- | --- | --- | --- | --- | --- |
| **Country** |  | **local providers** | **clinics offering single treatments** | **gender clinics offering most treatments** | **Additional free text responses** |  |
| **Argentina** |  | x | x |  | - “[since 2012] many trans-sensitive services have grown in the public system. These services usually provide HIV testing and care; hormone therapy and mental health [care]” - “local providers for hormone treatment and clinics for surgery” | - difficult access to transgender healthcare - health care access via “trans-friendly” clinics |
| **Australia** |  | x | x | x | - “Varies greatly across regions” - “public hospital-based clinics, private mental health professionals and primary clinics provide assessment and prescribe hormones. Surgery is done privately.” | - “gender centers” in some regions, in other regions only local providers |
| **Austria** |  | x | x |  | - “Mental healtcare for transgender persons is completely allocated in private practice settings“ - “Some endocrinological out-patient's clinics exist within gynaecological departments” - “hardly any collaboration between various disciplines, neither in academic settings nor other hospitals” | - single outpatient units in university and non-university hospitals, - small number of mental health professionals in private practice providing transgender health care |
| **Belgium** | - One specialized clinic for the whole country offering all treatments necessary for gender affirmation | x |  | x | - “Transgender health care is provided in three Universital Hospitals although only one University Hospital is offering Gender affirming surgery” - “some mental health care providers, endocrinologists and surgeons, who have a private practice and in some extent work together with the University Hospitals” | - two additional, smaller centers in Luik and Brussels - local providers offering mental health care and hormone treatment |
| **Brazil** |  | x | x | x | - “transgender related care is provided by public university hospital. Five from that time are currently running.” | - gender-affirming surgery is provided in specialized centers, single treatments offered in local hospitals - lack of experienced providers |
| **Canada** |  | x | x | x | - “Each province and territory in Canada has its own care delivery system but most are decentralized or a combination of both” | - one clinic in Montreal offering all treatment options - local centers offering single treatments |
| **Chile** |  | x | x |  | - “Transgender health care in Chile is provided in 7 public hospitals, just three of them work in a centralized way, including mental health team, surgery and hormone treatment” | - transgender health care procedures publicly founded - lack of specialized providers |
| **China** |  | x | x |  | - “Transgender health care in China is provided in different hospitals and NGOs. Only one hospital is centralized, providing diagnosis, mental health care, hormone treatment, and surgery.” | - five clinics offering gender-affirming treatment - one of them is multidisciplinary and offers a range of gender affirming treatments - access to transgender health care highly restricted - cost is not covered by public basic health insurance |
| **Colombia** |  | x | x |  | - “5 gender clinics at biggest hospitals at main capitals of the country. Mostly, in case of adults, endocrinologists tend to prescribe hormone treatment and recommend psychology and psychiatry evaluation simultaneously” - “In rural areas or other smaller cities there is no transgender health care” | - many transgender individuals are not aware of transgender health care services or cannot afford them |
| **Denmark** | - One specialized clinic for the whole country |  |  |  |  | - Since 2017 two centers (Copenhagen University Hospital, Aalborg University Hospital) that offer Gender-affirming treatment |
| **Finland** |  | x | x | x | - “four psychiatric clinics (2 adult psychiatry clinics and 2 clinics for 13-18 years old) at the University Hospitals in Helsinki and Tampere.” - “Genital surgery is centralized in one unit in Helsinki University Hospital.” - “Mental health care is provided (short therapy) in Helsinki Universal Hospitals Gender Clinic/adult psychiatry and in local public mental health care services” | - difficult access to transgender health care - psychiatric consultation in one of the gender clinics necessary |
| **France** |  | x | x | x | - “seven university public hospitals offering all necessary treatment options as well as by local health care professionals.” - “local health care professionals offer hormone treatment and mental health care.” - “functioning network between health care professionals from gender clinics and local providers” | - four university hospitals offering treatments |
| **Germany** |  | x | x | x | - “Hormonal treatment, Psychological support, surgical treatment, speech therapy, hair removal is offered at different places by different providers.” - “Transgender health care for children and adolescents in Germany is provided by five child and adolescent university centers as well as some non-university centers plus local health care professionals.” - “Medical treatment (puberty suppression and/or cross-sex hormones) is provided by some university centers as well as local pediatric endocrinologists.” | - one gender clinic - specialized providers in private practice - lack of access in rural areas |
| **Greece** |  | x | x |  | - “no gender health clinics existing and in public hospitals there are random outpatient clinics that provide mental health care or hormone therapy for gender diverse people.” - “transgender persons are seeking treatment in the private sector.” | - difficult access to transgender health care |
| **Hungary** |  | x | x |  | - “local Professionals offer mental health care and hormonal treatment.” - “Some surgeons perform gender affirmative genital surgery but has not got wide experience and could not offer phalloplasty options in general. We have only one university clinic who allowed to perform genital surgery in public system with only 10% insurance support.” - “Most of the patients are planning the surgeries abroad.” | - difficult access to transgender health care - lack of specialized providers - new law (2020) forbids change of legal status and name |
| **India** |  | x | x | x | - “currently […] only one transgender clinic which is under the Govt hospital settings.” - “thousands of Govt Hospitals which do provide services to Transgender [people]” - “Majority of Indian transgender [individuals] prefer going to private doctors which are apparently very expensive […]. | - lack of specialized providers in public hospitals - transgender people need to go to private providers - there is one free clinic in Kerala (only runs once a month out of a govt hospital) that offers treatment for depression and some gender affirming treatments (no surgeries) |
| **Indonesia** |  | x | x |  | - “Transgender health care in Indonesia is uncommon and generally provided by individual doctor.” - “No hospital that openly accept and provide transgender health care.” | - one clinic for LGBT health (but no gender-affirming treatments are offered) |
| **Iran** |  | x | x |  | - “Apart from the very clinic which is founded and run under my own authority, there are only anecdotal persons involved” | - few specialized clinics and local providers - lack of clinical knowledge regarding transgender health care |
| **Ireland** |  | x | x |  | - “one publicly funded centralized hospital based in the capital (Dublin)” - “2 other health professionals (Endo) in separate regions providing both a private and public service to trans clients” - “very limited access to gender affirming surgery, and so for those who would like to proceed with surgery we facilitate them with pre-surgical assessment and referral to their preferred centre in Europe” | - one publicly founded hospital offering transgender health care - no gender-affirming surgery in Ireland, but surgery outside Ireland is covered |
| **Israel** |  | x | x |  |  | - Feminizing genital surgery offered in one clinic - no masculinizing surgery due to the lack of experience |
| **Italy** |  | x | x | x | - “About 10 public hospitals throughout Italy. There is a network among centers.” | - Nine hospitals offering transgender health services |
| **Japan** |  | x | x |  | - “[…] few gender clinics offering SRS and hormone treatment.“ - “Some psychologists provide counseling to get a diagnosis.” | - Few clinics offering single transgender health services (HRT, GGAS) - high legal barriers to access transgender health care |
| **Mexico** |  | x | x |  | - “Some clinics with all treatments, but no coverage. Mostly local clinics for hormones, SRS is often too expensive” | - One clinic focusing on transgender health care free of charge (Mexico City) - Few private clinics offering single transgender health services (HRT, GGAS), |
| **The Netherlands** | - One specialized clinic for the whole country offering all treatments necessary for gender affirmation | x |  | x | - “one center that offers all treatment options for all ages. There is one center that offers care only to adults and one for children and adolescents (no surgery).” - “few endocrinologists outside the gender centres that offer hormone treatment.” | - Three specialized centers providing transgender health care (Amsterdam, Groningen, Leiden) |
| **New Zealand** |  | x | x |  | - “Limited public funding for a local private surgeon.“ - “Different providers in different parts of the country provide access to gender affirming hormones […]” | - Five centers and a few providers in private practice offering transgender health services - Education program in transgender health care |
| **Norway** |  | (x) |  | x | - “clinic in Oslo tries to be the only one to offer treatment for trans people” - “some local psychologists and endocrinologists try to offer treatment to, but the oslo clinic is trying to suppress us, as they think they are the only providers with proper knowledge” | - only one center providing transgender health services (Oslo) |
| **Pakistan** |  | x | x |  | - “a few special wards in hospitals that cater exclusively to transgender patients.” - “Transgender [individuals] have historically received medical care alongside other people in public and private health facilities.” | - Free health care for transgender individuals - no known health care structure for transgender health services so far - high barriers in access to transgender health care |
| **Peru** |  | x | x |  | - “focused only on transgender women as part of a technical guide for HIV prevention.” | - Transgender health services related to HIV care and only offered to trans woman - no service for transman so far |
| **Poland** |  | x | x |  | - “[Transgender health care] spread mostly to private practices and informal network between professionals.” - “Sexolog[y] Clinics, which also deliver services for other sexual concerns.” | - few sexology clinics and providers in private practice - Access restrictions to transgender health care due to the lack of legal gender recognition |
| **Portugal** |  | x | x |  | - “Transgender health care in Portugal is provided by 5 public hospitals offering mental health care and hormone treatment and only one surgery.” | - Five clinics offering psychological counselling and HRT - one clinic offering GGAS - Gender affirming treatment covered by insurance |
| **Russia** |  | x | x |  | - “Medical care for transgender people is not spelled out in legislation” - “transgender people may be denied assistance (for example, they will not be given a prescription for a hormonal drug) due to ignorance of the specifics.” | - Almost no possibilities to access transgender health care due to legal barriers and discrimination by health care providers |
| **Serbia** |  | x |  | x | - “[one center] offer[ing] complete care of transgender people, and includes psychiatrists, psychologists, endocrinologists, gynecologist, plastic surgeons and transgender surgeons.” | - one specialized clinic in Belgrad |
| **South Africa** |  | x | x |  | - “In the public health sector, some services are provided at central hospitals, e.g. mental health, endocrinology, surgery. […] not available in every province” - “In the private sector, some trans people access hormones from general practitioners.” - “Access to surgery is very limited with few skilled providers, long waiting lists in the public sector and exorbitant fees in the private sector” | - two specialized clinics offering transgender health services - few clinics offering single treatments |
| **Spain** |  | x | x |  | - “Public hospitals (not many) provide hormonal treatment and SRS” | - specialized outpatient clinics, - few clinics offering single treatments |
| **Sweden** | - Six specialized clinics for the whole country offering all treatments necessary for gender affirmation | x |  | x | - “3 university hospitals are providing the full range of procedures (including genital surgery) About 5 additional centres are providing part of the procedures (no genital surgery)” |  |
| **Switzerland** |  | x | x | x | - “ The University hospital in Basel is currently the only one offering all treatment options” - “Most trans people search individual and decentralized care” | - one specialized center (Basel) - few clinics providing single treatments (e.g., Zurich) - lack of specialized providers |
| **Turkey** |  | x | x |  | - “Local health care professionals offer hormone treatment and mental health care” | - few clinics providing single treatments - lack of specialized providers and transgender-specific knowledge |
| **U.K.** | - specialized gender identity service |  |  | x | - “8 regional Gender Identity Clinics, primarily led by Psychiatrists or Psychologists.” - “Scotland has 1 major clinic which runs 4 satellite clinic. Wales has 1 centralised clinic. Northern Ireland has a part-time clinical service for trans people.” - “clinics provide assessment and hormone therapies, and approve patients for gender surgery.” | - eight clinics offering transgender health care under the National Health Service (NHS) |
| **Ukraine** |  | x |  |  | - “psychiatric institutions provide diagnostic assessment as well as some single psychiatrists.” - “only few endocrinologists qualified in transgender hormone treatment” | - transgender health services only accessibly at practices of “sympathetic doctors” - high legal barriers to access transgender health services - lack of specialized providers |
| **U.S.** | - Transgender health care in the U.S. often in a patchwork of community clinics | x | x | x | - “Large diversity in settings within USA - both specialized gender clinics and academic and non-academic community health settings.“ - “most academic medical institutions offer some form of gender affirming health care.” - “Many options nationwide but often focused in urban areas” | - specialized centers in metropolitan areas (e.g., New York City, Boston, San Francisco) - clinics offering single treatments - specialized private providers - lack of resources in rural areas |

Table S2 Effects of (de-)centralized transgender health care delivery

|  |  |  | Statistics |
| --- | --- | --- | --- |
| **Health care setting affecting** | centralized health care delivery | decentralized health care delivery |  |
|  | N^o^ (%) | N^o^ (%) |  |
| involvement/ active contribution of the transgender community |  |  |  |
| Positively | 16 (34.0) | 21 (26.9) | χ^2^ (3, N=125)= 1.234 ; p=.745 |
| Negatively | 11 (23.4) | 18 (23.1) |  |
| Neither positively nor negatively | 13 (27.7) | 22 (28.2) |  |
| I cannot judge / I don’t know | 7 (14.9) | 17 (21.8) |  |
|  |  |  |  |
| professional exchange of expertise between institutions and providers of transgender health care |  |  |  |
| Positively | 16 (34.0) | 16 (20.5) | χ^2^ (3, N=125)= 4.823 ; p=.185 |
| Negatively | 7 (14.9) | 17 (21.8) |  |
| Neither positively nor negatively | 12 (25.5) | 30 (38.5) |  |
| I cannot judge / I don’t know | 12 (25.5) | 15 (19.2) |  |
|  |  |  |  |
| collaboration with non-medical community-linked institutions |  |  |  |
| Positively | 12 (25.5) | 17 (21.8) | χ^2^ (3, N=125)= 2.374 ; p=.498 |
| Negatively | 9 (19.1) | 20 (25.6) |  |
| Neither positively nor negatively | 18 (38.3) | 22 (28.2) |  |
| I cannot judge / I don’t know | 8 (17.0) | 19 (24.4) |  |
|  |  |  |  |
| collaboration with health insurances |  |  |  |
| Positively | 6 (12.8) | 4 (5.1) | χ^2^ (3, N=125)= 3.402 ; p=.334 |
| Negatively | 10 (21.3) | 25 (32.1) |  |
| Neither positively nor negatively | 15 (31.9) | 24 (30.8) |  |
| I cannot judge / I don’t know | 16 (34.0) | 25 (32.1) |  |
|  |  |  |  |
| Personal preference regarding health care delivery setting |  |  |  |
| centralized | 24 (51.1) | 31 (39.7) | χ^2^ (2, N=125)= 4.642 ; p=.098 |
| decentralized | 11 (23.4) | 33 (42.3) |  |
| I cannot judge / I don’t know | 12 (25.5) | 14 (17.9) |  |
|  |  |  |  |

Table S4 Factors derived from the free text responses on centralized health care (working in centralized setting)

| **Pro** | **Con** |
| --- | --- |
| Interdisciplinarity (9) | Longer travel time / difficult access for patients who live further away (8) |
| Ease of communication between staff members involved (4) | Long waiting lists (5) |
| better expertise and competence/ team experienced in transgender issues (8) | Less possibilities to look for a second opinion/ less choice of providers (6) |
| Possibility to conduct training/have better trained professionals (3) | Gatekeeping (2) |
| Higher volume of patients leads to greater experience (2) | Protocols (1) |
| Well defined protocols of care (1) | Patients may be “forced” to seek care in the centralized clinic even if they don’t want it (would prefer to stick to a known local provider; don’t want to feel like they need “special” care; etc.) (1) |
| Surgeons have better knowledge (1) | Difficulty o get care for non transgender related medical issues (general health) due to stigma/lack of knowledge in the rest of the medical care system (2) |
| Comprehensive patient care (5) | Can be more detached from communities of interest / know nothing about the community and background (3) |
| One stop shop/Patients can get multiple appointments on the same day (good for patients with transportation issues) (3) | More vulnerable to budget cuts and to the political environment (which can be more or less trans friendly) (3) |
| Consistency of care (Patient doesn’t have to deal with multiple professionals in multiple settings and repeating information or procedures) (3) | Difficult communication with health centers/ primary care closer to the patient (3) |
| Transgender friendly environment (2) | You need a huge clinic to offer that many treatments (1) |
| Better communication also makes sharing patients’ preferences/wishes easier (1) | Complex organization (1) |
| Easy access to care (1) | None (1) |
| Patients have easier access to the whole team (1) | More anonymous (1) |
| The possibility to conduct academic research (4) |  |
| More support to obtain gender affirming treatment (such as hormones) from insurances or from states (1) |  |
| Self-reliance before self-affirmation (1) |  |
| Free of charge (1) |  |

Table S5 Factors derived from the free text responses on centralized health care (working in decentralized setting)

| **Pros** | **Cons** |
| --- | --- |
| Interdisciplinarity (11) | Longer travel time / difficult access for patients who live further away (14) |
| Offer more robust services like speech therapy and hair removal (1) | Long waiting lists / waiting time for patients (6) |
| Patients more likely to access different services (1) | Future social exclusion due to difficult access (1) |
| Easier consultation for surgery (1) | Difficult access to general healthcare (1) |
| Centralized records and access (1) | Less possibilities to look for a second opinion/ less choice of providers (10) |
| Specialized care/better expertise (11) | Risk of abuse of the exclusive position by the institution, i.e. by gatekeeping or monopoly of treatment (5) |
| Standardized care / standard protocols (3) | Only one possibility for surgical interventions / one surgeon (even if they use techniques that are not up-to date or are marginally competent) (1) |
| Good clinical practice (GCP) (1) | Less practitioners involved in healthcare (1) |
| Good for improving experience (1) | If in conflict with anybody of the personnel, there might be fear of this conflict spreading to the whole care system (1) |
| Easier access (10) | Few clinics where centralized care would be possible/could be offered (1) |
| One stop shop / treatment in one place (9) | Possible institutionalization (1) |
| Comprehensive care (5) | Control (1) |
| Reduced stigma (2) | Protocols are inflexible, could lead to not taking personal needs into account (4) |
| Very good for kids and teens who need follow ups and treatment teams (1) | Lack of high degree of qualification of the staff (2) |
| Easy to identify and reduce participants’ burden (1) | Not enough clinics in a huge country (1) |
| Fast track through transition (1) | Could be expensive in some areas where there are not that many trans people to treat (1) |
| Continuity of care (1) | Increased burden on the facilities due to their small number (1) |
| Short waiting lists (1) | At risk for budget cuts (1) |
| Easy communication (1) | Clients report being "lost" in the system. (1) |
| Research possibilities (3) | Less privacy / confidentiality risk (2) |
| Can work if there are satellite offices throughout the country/province/state (1) | History of pathologizing transgender people(1) |
| Cost savings for the institution (1) | Shouldn't have to be a special clinic to visit! (1) |
| Location (1) | None (1) |
| You can know and manage the trans situation proposing public policies. (1) |  |

Table S6 Factors derived from the free text responses on decentralized health care (working in centralized setting)

| **Pros** | **Cons** |
| --- | --- |
| Reduced travel requirements (10) | Less professional competence or experience / lack of professional training or difficulty in training the necessary professionals (10) |
| Shorter waiting lists / faster access (5) | possibly less knowledge of the treatment options offered by other disciplines/patients may not get all the care needed (3) |
| Easier access / availability (3) | More traveling to and between providers (2) |
| More care options/choice of providers (6) | Less consistency in care (3) |
| Knowledge of and better involvement in the community (4) | Difficult to organize research (2) |
| Networking (2) | Less effective communication between professionals involved in the same person's care (1) |
| Easier communication with primary care and other med. services the patient uses (1) | Hard to organize and coordinate appointments and treatment at multiple centers/sites (3) |
| Affordable (1) | Less interdisciplinarity (3) |
| Self and general self-affirmation (1) | Less agreement btw professionals related to the person they are consulting (1) |
| hugh countries need more centers to cover all transgender population, like Germany, Australia or US... (1) | Costly (1) |
| More trained staff/services (2) | Inconvenient for consumers (1) |
| Better confidentiality (1) | Easier to slip to the cracks with no follow up (1) |
| Lower cost for healthcare system (1) | Insurance may be an issue at different locations (1) |
| More knowledge about transgender care in the general health care system (1) | Long waiting lists (1) |
| Social input (1) | Surgical procedures may need to be based in a main center (1) |
| Contrary (1) | more complex clinical governance (1) |
| None (3) | physical and therapeutic resources available (1) |
|  | minority stress, hipersensibility to rejection (1) |

Table S7 Factors derived from the free text responses on decentralized health care (working in decentralized setting)

| **Pros** | **Cons** |
| --- | --- |
| Easier access / availability (16) | Professionals’ lack of experience, training and knowledge (9) |
| Less travel requirements (9) | Difficulty in finding certain specialists or resources (8) |
| Access to individualized care / centered on patients’ needs (7) | Variance in quality of care (3) |
| Faster access to care / shorter waiting lists (1) | No standardized care (1) |
| More care options/choice of providers (14) | Not providing state of the art knowledge/care, not applying newest research data (1) |
| It is possible to get different opinions (2) | Difficult communication btw providers / Harder to coordinate services (9) |
| Less likely to limit the number of care providers (1) | Fragmentation of care (5) |
| Independence (1) | Patient has the responsibility of coordinating their own treatment (3) |
| Good for routine checks (1) | Less access, especially when finances or insurance are limited (6) |
| Mistakes can be corrected by other providers (1) | Less organized (1) |
| Knowledge and understanding of the community (2) | Requires multiple appointments in different places (2) |
| Leads to less stigma as it is similar to routine medical check-ups/ normalizes transitioning, in contrast to consulting a centralized institution (2) | Little contact with surgical care providers (1) |
| More comfort for the patient (1) | Resources clustered in big cities / capitals (1) |
| More informed consent (1) | More chances of encountering stigma (2) |
| Confidentiality ensured (1) | Less comfort/ more stress for the patients (2) |
| None (1) | Increased cost to the system (3) |

Table S8 Grey literature search

| **Argentina** | Difficult access to health care:  Socías, M. E., Marshall, B. D. L., Arístegui, I., Romero, M., Cahn, P., Kerr, T. & Sued, O. (2014). Factors associated with healthcare avoidance among transgender women in Argentina. International Journal for Equity in Health, 13(1), 81. <https://doi.org/10.1186/s12939-014-0081-7>  Braz, C. (2019). Vidas que esperam? Itinerários do acesso a serviços de saúde para homens trans no Brasil e na Argentina [Lives on hold? Itineraries in access by trans men to health services in Brazil and Argentina]. Cadernos de saude publica, 35(4), e00110518. <https://doi.org/10.1590/0102-311X00110518>  Access to health care only through "trans-friendly clinics ":  Breaking down barriers to healthcare access for transgender people in Argentina - PAHO/WHO \| Pan American Health Organization. (30. September 2020). <https://www.paho.org/en/news/17-4-2018-breaking-down-barriers-healthcare-access-transgender-people-argentina>  PAHO, JSI & WPATH. Blueprint for the Provision of Comprehensive Care for Trans Persons and Their Communities in the Caribbean and Other Anglophone Countries. <https://www.paho.org/hq/dmdocuments/2014/2014-cha-blueprint-comprehensive-anglo-countries.pdf> |
| --- | --- |
| **Australia** | Two gender clinics; less populated areas only have private providers:  Telfer, M., Tollit, M. & Feldman, D. (2015). Transformation of health-care and legal systems for the transgender population: The need for change in Australia. Journal of paediatrics and child health, 51(11), 1051–1053. https://doi.org/10.1111/jpc.12994  Map with gender clinics and local providers:  Trans Health Australia. (5. Oktober 2020). Medical Services Map - Trans Health Australia. https://www.transhealthaustralia.org/index.php/medical-services/medical-map |
| **Austria** | Lists with outpatient departments and local providers:  Transambulanzen – TTA – Transgender Team Austria. (5. Oktober 2020). <http://transgender-team.at/infos/transambulanzen/>  Chirurgen \| Urologen \| Gynäkologen – TTA – Transgender Team Austria. (5. Oktober 2020). <http://transgender-team.at/chirurgen/>  PsychotherapeutInnen – TTA – Transgender Team Austria. (5. Oktober 2020). <http://transgender-team.at/infos/psychotherapeutinnen/>  Most outpatient departments offer hormone replacing therapy and gender-affirming surgery:  Transgender-Ambulanz. (5. Oktober 2020). <https://frauenheilkunde.meduniwien.ac.at/gynendo/patientinneninformationen/abklaerung-von-hormonellen-stoerungen/transgender-ambulanz/>  The road to transitioning in Austria:  TransX. (5. Oktober 2020). <https://www.transx.at/Pub/Medizinisches.php>  Trans-specific health care services provided by the public health care system:  Güldenring, A., van Trotsenburg, M. & Flütsch, N. (2019). Queering Medicine – Dringlichkeit einer bedürfnisorientierten und evidenzbasierten Transgendergesundheitsversorgung. Journal für Klinische Endokrinologie und Stoffwechsel, 12(3), 84–94. <https://doi.org/10.1007/s41969-019-00075-8>  The transgender center in Innsbruck has an interdisciplinary approach and offers all gender-affirming treatments:  tirol kliniken gmbh. (5. Oktober 2020). Transgender Center Innsbruck. <https://www.tirol-kliniken.at/page.cfm?vpath=standorte/landeskrankenhaus-innsbruck/weitere-einrichtungen/transgender-center-innsbruck> |
| **Belgium** | Three gender clinics in Luik, Brussels, and Ghent (clinic also has a specialized team for trans minors):  Elaut, E. (2014). Transgender health care in Belgium. https://epath.eu/wp-content/uploads/2014/07/Transgender-health-care-in-Belgium_Els-Elaut_20150313.pdf  List with local providers:  Transgender Infopunt \| Zorgaanbod \|. (6. Oktober 2020). <http://transgenderinfo.be/l/zorgaanbod/>   - Seite ist nicht auf Englisch, ist aber ziemlich selbsterklärend.   Some hospitals offer gender-affirming treatments (Page 78):  van den Brink, M. & Dunne, P. (2018). Trans and intersex equality rights in Europe – a comparative analysis. https://ec.europa.eu/info/sites/info/files/trans_and_intersex_equality_rights.pdf |
| **Brazil** | Gender-affirming surgeries are covered by the public health care system:  The Associated Press (18. August 2007). Brazil: Free Sex-Change Operations. The New York Times. <https://www.nytimes.com/2007/08/18/world/americas/18briefs-FREESEXCHANG_BRF.html>  11 clinics offer gender-affirming surgeries (locations are not specified):  Schwartz Benzaken, A. (2018). Transgender legislation and strategic information on transpeople in Brazil. https://hivpreventioncoalition.unaids.org/wp-content/uploads/2018/12/Brazil-transgender-legislation.pdf  Difficult access to transgender-specific health care:  Lima, R. R. T. de, Flor, T. B. M., Silva, A. B. & Noro, L. R. A. (2020). Health care for transgender people in Brazil: a systematic review protocol. <https://doi.org/10.21203/rs.3.rs-58007/v1>  Gender-affirming surgeries offered by centralized and local clinics, but lack of experienced providers:  Tagliamento, G. & Paiva, V. (2016). Trans-Specific Health Care: Challenges in the Context of New Policies for Transgender People. Journal of Homosexuality, 63(11), 1556–1572. <https://doi.org/10.1080/00918369.2016.1223359> |
| **Canada** | List and map of gender-affirming care options:  Publicly funded gender-affirming medical care in Canada. (2018). <http://www.ufcw.ca/templates/ufcwcanada/images/media/posters/Publicly_Funded_Transition_Posters/SRS_genderAssignment_8.5x11_EN_2018.pdf>  Local centers help with search and coordination of gender-affirming care:  Who We Are. (6. Oktober 2020). http://www.phsa.ca/transcarebc/about/who-we-are  Local clinics offering single gender-affirming treatments:  Klinic Community Health. (6. Oktober 2020). Trans Health Klinic - Klinic Community Health. <https://klinic.mb.ca/health-care/transgender-health-klinic/>  Clinic 554 - Our Care. (21. September 2015). <http://www.clinic554.ca/care.html>  Halifax Sexual Health Centre. (6. Oktober 2020). Transgender Health. <http://hshc.ca/transgender-health/>  Trans Health. (5. Oktober 2020). Trans Health Clinics - Trans Health. <http://www.trans-health.com/clinics/>  The only clinic that offers all gender-affirming surgeries and treatments:  GRS Montréal. (6. Oktober 2020). GrS Montréal \| Chirurgies d'affirmation de genre à Montréal. <https://www.grsmontreal.com/> |
| **Chile** | Gender-affirming treatments are covered by the public health care system:  National LGBTQ Task Force. (2012). Progress on transgender health care in Chile - National LGBTQ Task Force. <https://www.thetaskforce.org/progress-on-transgender-health-care-in-chile/>  Transgender-specific health care is often provided by inexperienced personnel:  Zapata Pizarro, A., Díaz Díaz, K., Barra Ahumada, L., Maureira Sales, L., Linares Moreno, J. & Zapata Pizarro, F. (2019). Atención de salud de personas transgéneros para médicos no especialistas en Chile [Healthcare of transgenders by non-specialists in Chile]. Revista medica de Chile, 147(1), 65–72. <https://doi.org/10.4067/S0034-98872019000100065> |
| **China** | Difficult access and high costs for gender-affirming treatments:  Zhu, X., Gao, Y., Gillespie, A., Xin, Y., Qi, J., Ou, J., Zhong, S., Peng, K., Tan, T., Wang, C. & Chen, R. (2019). Health care and mental wellbeing in the transgender and gender-diverse Chinese population. The Lancet Diabetes & Endocrinology, 7(5), 339–341. https://doi.org/10.1016/S2213-8587(19)30079-8  Access to gender-affirming surgeries is restricted by a set of guidelines deciding which patients qualify for treatment:  Jiang, H., Wei, X., Zhu, X., Wang, H. & Li, Q. (2014). Transgender patients need better protection in China. The Lancet, 384(9960), 2109–2110. <https://doi.org/10.1016/S0140-6736(14)62372-2>  Five clinics offer hormone therapy and gender-affirming surgeries, but the cost is not covered by the public health care system:  Liu, Y., Xin, Y., Qi, J., Wang, H., Hong, T., Yang, X., ... & Pan, B. (2020). The Desire and Status of Gender-Affirming Hormone Therapy and Surgery in Transgender Men and Women in China: A National Population Study. *The Journal of Sexual Medicine*.  Lack of care options leads to patients self-medicating:  Liu, Y., Xin, Y., Qi, J., Wang, H., Hong, T., Yang, X., Li, B., Chang, X., Knudson, G., Zhao, Z. & Pan, B. (2020). The Desire and Status of Gender-Affirming Hormone Therapy and Surgery in Transgender Men and Women in China: A National Population Study. The Journal of Sexual Medicine. Advance online publication. <https://doi.org/10.1016/j.jsxm.2020.07.081>  One interdisciplinary hospital (Peking) providing all treatments:  Transgender people in China risk their lives with dangerous self-surgery. (6. Oktober 2020). <https://www.amnesty.org/en/latest/news/2019/05/china-transgender-dangerous-self-surgery/>  Some local providers offer gender reassignment surgeries:  Floris, L. P. (9. Juli 2019). Being transgender in China: activist on her struggles, and surgeon on his patients' biggest challenge. South China Morning Post. <https://www.scmp.com/lifestyle/health-wellness/article/3017823/transgender-china-trans-activist-her-struggles-and>  Reasons for the difficult access to gender-affirming care:  Dahlkemper, J., Diaz, B., Lawanson, F., Messina, J. & Morris, E. (2019). TRANSGENDER HEALTH CARE IN CHINA: Final Report & Recommendations. <https://cnlgbtdata.com/files/uploads/2020/04/Transgender_Helathcare_in_China.pdf> |
| **Colombia** | Lack of information for trans people about gender-affirming treatment possibilities and difficulty affording them:  Aguayo-Romero, R. A., Reisen, C. A., Zea, M. C., Bianchi, F. T. & Poppen, P. J. (2015). Gender Affirmation and Body Modification Among Transgender Persons in Bogotá, Colombia. The international journal of transgenderism, 16(2), 103–115. <https://doi.org/10.1080/15532739.2015.1075930>  Access to gender-affirming health care is impossible without undergoing a process of pathologization:  OutRight Action International. (2016). The Situation for Trans People in Colombia. <https://outrightinternational.org/content/situation-trans-people-colombia> |
| **Denmark** | One centralized gender clinic in Kopenhagen offers all gender-affirming treatments (hormone therapy can be continued by local providers):  The Post. (2016). Demand for transgender medical care in Denmark rising. http://cphpost.dk/?p=76799  Access to gender-affirming care difficult due to a lack of choice of medical providers:  Murmur » "The state is killing my identity". <http://murmur.dk/the-state-is-killing-my-identity/>  A second gender clinic was opened in 2017 at the Aalborg University Hospital and is offering all gender-affirming treatments:  Dietz, C. (2020). Jurisdiction in Trans Health. Journal of Law and Society, 47(1), 60–86. https://doi.org/10.1111/jols.12212 |
| **Finland** | Difficult access to transgender health care:  THE CRUEL, INHUMAN AND DEGRADING TREATMENT OF TRANS AND INTERSEX PEOPLE IN FINLAND. (2016). https://tbinternet.ohchr.org/Treaties/CAT/Shared%20Documents/FIN/INT_CAT_CSS_FIN_25540_E.pdf  Gender-affirming treatments only possible after a psychiatric consultation from the gender clinics in Helsinki or Tampere:  Staff, N. N. (8. März 2018). Life In Limbo: How Finland Is Failing On Gender Identity Rights. News Now Finland. <https://newsnowfinland.fi/politics/life-in-limbo-how-finland-is-failing-on-gender-identity-rights>  Gender clinics at the University Hospitals in Helsinki and Tampere offer gender-affirming treatments:  Trasek ry. (2011). In English. <http://trasek.fi/in-english/>  The two gender clinics have an interdisciplinary approach:  Medical treatments for gender dysphoria that reduces functional capacity in transgender people – recommendation. (2020). https://palveluvalikoima.fi/documents/1237350/22895838/Summary+transgender.pdf/2cc3f053-2e34-39ce-4e21-becd685b3044/Summary+transgender.pdf |
| **France** | Six medicals teams and 254 local providers mentioned in the Methods section:  Giami, A. & Beaubatie, E. (2014). Gender identification and sex reassignment surgery in the trans population: a survey study in France. Archives of Sexual Behavior, 43(8), 1491–1501. <https://doi.org/10.1007/s10508-014-0382-3>  Four interdisciplinary university clinics:  Solutions, S. (2. Oktober 2020). Transgender Health Care in Europe: France. <https://programme.exordo.com/epath2017/delegates/presentation/170/> |
| **Germany** | List with local providers:  Adressen – TransMann e.V.®. (6. Oktober 2020). <https://transmann.de/adressen/>  One interdisciplinary gender clinic offering all gender-affirming treatments:  Eyssel, J., Koehler, A., Dekker, A., Sehner, S. & Nieder, T. O. (2017). Needs and concerns of transgender individuals regarding interdisciplinary transgender healthcare: A non-clinical online survey. PloS one, 12(8), e0183014. <https://doi.org/10.1371/journal.pone.0183014>  Local clinics offering some gender-affirming treatments:  Xenia (13. September 2015). Übersicht der Kliniken, die in Deutschland, die Geschlechtsangleichende Operation durchführen. Gendertreff. <https://gendertreff.de/2015/09/13/uebersicht-der-kliniken-die-in-deutschland-die-geschlechtsangleichende-operation-durchfuehren/> |
| **Greece** | Difficult access to transgender health care; lack of experienced providers:  Halkitis, P. N., Valera, P., & Kantzanou, M. (2018). Deterioration in social and economic conditions in Greece impact the health of LGBT populations: A call to action in the era of Troika. Psychology of Sexual Orientation and Gender Diversity, 5(4), 503–507.  Trans people are "invisible " in the health care system:  Giannou, D., & Ioakimidis, V. (2020). ‘Neither invisible nor abnormal!’Exploring the invisibility and pathologisation of LGBT people in the Greek National Health System. Critical Social Policy, 40(3), 370-388. <https://doi/10.1177/0261018319840187>  Since 2017 legal recognition:  Greek transgender community hopes new law will improve lives (10. Oktober 2017). Associated Press. <https://apnews.com/article/165d73650fd940b981b3b3f5cad56022>  Unclear if gender-affirming treatments are available (page 78):  van den Brink, M. & Dunne, P. (2018). Trans and intersex equality rights in Europe – a comparative analysis. https://ec.europa.eu/info/sites/info/files/trans_and_intersex_equality_rights.pdf |
| **Hungary** | Gender-affirming care possible, 10% of costs provided by the national health insurance (page 78-79):  van den Brink, M. & Dunne, P. (2018). Trans and intersex equality rights in Europe – a comparative analysis. https://ec.europa.eu/info/sites/info/files/trans_and_intersex_equality_rights.pdf  Legal recognition has been denied by a new law:  Euronews (1. Juni 2020). Hungary's new anti-transgender law is threatening to set hard-won LGBT rights back decades ǀ View. Euronews. <https://www.euronews.com/2020/06/01/hungary-s-new-anti-transgender-law-is-threatening-to-set-hard-won-lgbt-rights-back-decades>  Lack of experienced medical providers:  Human Rights First. (4. Oktober 2020). Transgender Day of Awareness Guest Blog: Living in Hungary as a Trans Person. <https://www.humanrightsfirst.org/blog/transgender-day-awareness-guest-blog-living-hungary-trans-person>  Lack of medical standards for transgender health care and lack of specialized and experienced providers:  LGBTQI RIGHTS IN HUNGARY: A joint submission by the Hungarian LGBT Alliance, Transvanilla Transgender Association, Háttér Society, and Labrisz Lesbian Association. (2018). https://tbinternet.ohchr.org/Treaties/CCPR/Shared%20Documents/HUN/INT_CCPR_CSS_HUN_30243_E.pdf |
| **India** | Discrimination in access to health care:  Ming, L. C., Hadi, M. A. & Khan, T. M. (2016). Transgender health in India and Pakistan. The Lancet, 388(10060), 2601–2602. <https://doi.org/10.1016/S0140-6736(16)32222-X>  One project for increasing access to health care:  Shaikh, S., Mburu, G., Arumugam, V., Mattipalli, N., Aher, A., Mehta, S. & Robertson, J. (2016). Empowering communities and strengthening systems to improve transgender health: outcomes from the Pehchan programme in India. Journal of the International AIDS Society, 19(3 Suppl 2), 20809. <https://doi.org/10.7448/IAS.19.3.20809>  Lack of adequate mental health care for trans people:  Kalra, G. (2012). Hijras : the unique transgender culture of India. International Journal of Culture and Mental Health, 5(2), 121–126. <https://doi.org/10.1080/17542863.2011.570915>  One gender clinic in Kerala that offers treatment for depression and some gender-affirming treatments, only open one day a month:  BBC News. (4. Oktober 2020). The clinic helping India's transgender people - BBC News. <https://www.bbc.com/news/av/world-asia-india-41193417>  Many trans people choose private providers for gender-affirming treatments, to avoid deficient care:  Swaddle, T. (5. Oktober 2019). What a Transgender‑Friendly Health Care System Would Look Like. The Swaddle. <https://theswaddle.com/what-a-transgender-friendly-health-care-system-would-look-like/> |
| **Indonesia** | Project for improving mental health (inclusive for LGBT people and other minorities):  Inside Indonesia. (4. Oktober 2020). Building bridges for all - Inside Indonesia. <https://www.insideindonesia.org/building-bridges-for-all>  One clinic offers health care to LGBT people but does not offer gender-affirming treatments:  POZ. (2011). Indonesia Opens First Gay, Transgender Health Clinic. <https://www.poz.com/article/indonesia-hiv-clinic-21443-6352> |
| **Iran** | Transgender-specific gender clinics and local providers offer gender-affirming treatments:  Shirdel-Havar, E., Steensma, T. D., Cohen-Kettenis, P. T. & Kreukels, B. P. C. (2019). Psychological symptoms and body image in individuals with gender dysphoria: A comparison between Iranian and Dutch clinics. The international journal of transgenderism, 20(1), 108–117. <https://doi.org/10.1080/15532739.2018.1444529>  Providers' experience and treatment quality is uncertain:  Welle, D. (4. Oktober 2020). How Iran's anti-LGBT policies put transgender people at risk \| DW \| 28.04.2020. Deutsche Welle (www.dw.com). <https://www.dw.com/en/how-irans-anti-lgbt-policies-put-transgender-people-at-risk/a-53270136> |
| **Ireland** | Only access to hormone therapy, surgeries in other countries are paid for by the public health care system (page 78-79):  van den Brink, M. & Dunne, P. (2018). Trans and intersex equality rights in Europe – a comparative analysis. https://ec.europa.eu/info/sites/info/files/trans_and_intersex_equality_rights.pdf  One publicly funded hospital and local providers offer gender-affirming treatments:  Ruari-Santiago Mcbride. (2011). Healthcare Issues for Transgender People Living in Northern Ireland. <https://www.researchgate.net/profile/ruari_santiago_mcbride/publication/275026560_healthcare_issues_for_transgender_people_living_in_northern_ireland> |
| **Israel** | FtM surgeries are not available due to a lack of experienced personnel; MtF surgeries are offered in one clinic; access to surgery is decided by a national committee:  Sex Reassignment Surgery. (4. Oktober 2020). Sex Reassignment Surgery. <https://www.health.gov.il/English/Services/Citizen_Services/SexReassignmentSurgery/Pages/default.aspx> |
| **Italy** | Gender-affirming treatments are possible, unclear if funded by the public health care system:  van den Brink, M. & Dunne, P. (2018). Trans and intersex equality rights in Europe – a comparative analysis. https://ec.europa.eu/info/sites/info/files/trans_and_intersex_equality_rights.pdf  Six hospitals and clinics that offer gender-affirming treatments:  Caldarera, A. & Pfäfflin, F. (2011). Transsexualism and Sex Reassignment Surgery in Italy. International Journal of Transgenderism, 13(1), 26–36. <https://doi.org/10.1080/15532739.2011.605341>  Nine gender clinics that offer gender-affirming treatments:  Vitelli, R., Scandurra, C., Pacifico, R., Selvino, M. S., Picariello, S., Amodeo, A. L., Valerio, P. & Giami, A. (2017). Trans identities and medical practice in Italy: Self-positioning towards gender affirmation surgery. Sexologies, 26(4), e43-e51. <https://doi.org/10.1016/j.sexol.2017.08.001>  List of 21 centers and organizations that offer health care (at least mental health care) to trans people:  Where to find help! \| ILGA-Europe. (4. Oktober 2020). <https://ilga-europe.org/mental-health/help> |
| **Japan** | Gender clinics providing gender-affirming treatments:  Martínez Nieto, J. (2018). The situation of transgender people in Japan. https://ddd.uab.cat/pub/tfg/2018/201947/TFG_jmartineznieto.pdf  Difficulty in obtaining legal gender recognition:  Human Rights Watch. (2019). "A Really High Hurdle". <https://www.hrw.org/report/2019/03/19/really-high-hurdle/japans-abusive-transgender-legal-recognition-process> |
| **Mexico** | One clinic in Mexico City offering most gender-affirming treatments:  Pons Rabasa, A. (2016). From Representation to Corposubjectivation. TSQ: Transgender Studies Quarterly, 3(3-4), 388–411. https://doi.org/10.1215/23289252-3545119 (TSQ: Transgender Studies Quarterly, 3(3-4), 388-411).  The clinic in Mexico City offers mental health care, hormone therapy, and STI treatments:  Víctor Manuel Enriquez-Estrada. (2019). Health care, human rights and citizenship: Issues faced by trans people in Mexico. https://www.researchgate.net/publication/337590983_Health_care_human_rights_and_citizenship_Issues_faced_by_trans_people_in_Mexico  Access to gender-affirming surgeries and other treatments is difficult:  Lozano-Verduzco, I. & Melendez, R. (2019). Transgender individuals in Mexico: exploring characteristics and experiences of discrimination and violence. Psychology & Sexuality, 1–13. https://doi.org/10.1080/19419899.2019.1698449 (Psychology & Sexuality, 1-13).  Another clinic offering some of the gender-affirming surgeries:  Qunomedical. (4. Oktober 2020). Mexico Transgender Center at Green & Health Hospital Tijuana. <https://www.qunomedical.com/en/mexico-transgender-center-at-green-health-hospital-tijuana>  Only in the clinic in Mexico City are some services covered by the public health care system (only psychological and hormone treatments):  Global Press Journal. (2016). Transgender People in Mexico City Resort to Dangerous Unsupervised Procedures. <https://globalpressjournal.com/americas/mexico/transgender-people-mexico-city-resort-dangerous-unsupervised-procedures/> |
| **Netherlands** | Specialized trans health care offered in gender clinics (page 79):  van den Brink, M. & Dunne, P. (2018). Trans and intersex equality rights in Europe – a comparative analysis. https://ec.europa.eu/info/sites/info/files/trans_and_intersex_equality_rights.pdf  Three gender clinics offering gender-affirming treatments:  Sharpe, N. (2017). Being Transgender and Seeking Healthcare in the World's Most Liberal City. Independent Study Project (ISP) Collection. <https://digitalcollections.sit.edu/isp_collection/2649> |
| **New Zealand** | Program for training new specialized care providers:  Gamble Blakey, A. & Treharne, G. J. (2019). Overcoming Barriers to Transgender Healthcare Education in Aotearoa New Zealand. New Zealand Journal of Educational Studies, 54(2), 357–366. <https://doi.org/10.1007/s40841-019-00143-2>  Five clinics and local providers, offering different arrays of gender-affirming treatments:  Delahunt, J. W., Denison, H. J., Kennedy, J., Hilton, J., Young, H., Chaudhri, O. B. & Elston, M. S. (2016). Specialist services for management of individuals identifying as transgender in New Zealand. The New Zealand medical journal, 129(1434), 49–58. |
| **Norway** | One center legally allowed to diagnose and treat trans people:  van der Ros, J. (2017). The Norwegian State and Transgender Citizens: A Complicated Relationship. World Political Science, 13(1), 123–150. <https://doi.org/10.1515/wps-2017-0003>  The same centralized clinic confirmed here:  Sørlie, A. (2019). The Right to Trans-Specific Healthcare in Norway: Understanding the Health Needs of Transgender People. Medical law review, 27(2), 295–317. https://doi.org/10.1093/medlaw/fwy029 |
| **Pakistan** | Discrimination and inequality in access to health care (both specialized and generalized):  Ming, L. C., Hadi, M. A. & Khan, T. M. (2016). Transgender health in India and Pakistan. The Lancet, 388(10060), 2601–2602. <https://doi.org/10.1016/S0140-6736(16)32222-X>  Trans health care covered by the public health care system:  Bollinger, A. (22. Januar 2020). Pakistan gives free health care to transgender people. LGBTQ Nation. <https://www.lgbtqnation.com/2020/01/pakistan-gives-free-health-care-transgender-people/>  A specialized health care infrastructure for trans health care is still lacking:  Khan, A. & Shekhani, S. (28. März 2019). Are doctors ready for the medical needs of the transgender community? DAWN.COM. <https://www.dawn.com/news/1472414> |
| **Peru** | HIV prevention and gender-affirming treatments are offered to trans women:  Reisner, S. L., Perez-Brumer, A. G., McLean, S. A., Lama, J. R., Silva-Santisteban, A., Huerta, L., Sanchez, J., Clark, J. L., Mimiaga, M. J. & Mayer, K. H. (2017). Perceived Barriers and Facilitators to Integrating HIV Prevention and Treatment with Cross-Sex Hormone Therapy for Transgender Women in Lima, Peru. AIDS and Behavior, 21(12), 3299–3311. <https://doi.org/10.1007/s10461-017-1768-8>  The same health care offer for trans women is confirmed here:  Institute, The Fenway (9. Januar 2017). The Fenway Institute Helps Develop Model Of HIV Care For Transgender Women In Peru. Fenway Health: Health Care Is A Right, Not A Privilege. <https://fenwayhealth.org/the-fenway-institute-helps-develop-model-of-hiv-care-for-transgender-women-in-peru/> |
| **Poland** | One gender clinic and local providers offer gender-affirming treatments:  Bartosz, G. & Marta, D. (2019). Transgender healthcare in Poland. http://epath.eu/wp-content/uploads/2019/04/BartoszGrabski_Poland.pdf  Access to transgender health care is difficult:  Makarewicz-Marcinkiewicz, A. (2019). Resisting Gender Recognition in Poland: A Process of Social Exclusion. Polish Political Science Review, 7(2), 19–31. <https://doi.org/10.2478/ppsr-2019-0011>  Legal recognition is difficult and the public health care system does not cover gender-affirming treatments:  Smiley, A., Burgwal, A., Orre, C. & Summanen, E. (2017). Overdiagnosed but Underserved.: Trans Healthcare in Georgia, Poland, Serbia, Spain, and Sweden: Trans Health Survey. <https://tgeu.org/wp-content/uploads/2017/10/Overdiagnosed_Underserved-TransHealthSurvey.pdf> |
| **Portugal** | Five gender clinics offer psychological care and hormone therapy; one also offers gender-affirming surgeries; the public health care system covers gender-affirming treatments:  Hilário, A. P. (2020). Rethinking trans identities within the medical and psychological community: a path towards the depathologization and self-definition of gender identification in Portugal? Journal of Gender Studies, 29(3), 245–256. https://doi.org/10.1080/09589236.2018.1544066 (Journal of Gender Studies, 1-12). |
| **Russia** | Access to trans-specific care nearly impossible due to pathologization by the law and by the providers:  Dmitry, I. (2017). Transgender Health Care in Russia. http://epath.eu/wp-content/uploads/2018/06/transgender-healthcare-in-Russia.pdf  Trans people are not socially accepted; their access to transgender health care is dependent on a psychiatric diagnosis:  Smith, G. (8. August 2019). What's it like to be transgender in Russia? LGBTQ Nation. <https://www.lgbtqnation.com/2019/08/whats-like-transgender-russia/> |
| **Serbia** | One clinic offers gender-affirming treatments after psychiatric diagnosis; costs are not covered by public health care; access is difficult:  Smiley, A., Burgwal, A., Orre, C. & Summanen, E. (2017). Overdiagnosed but Underserved.: Trans Healthcare in Georgia, Poland, Serbia, Spain, and Sweden: Trans Health Survey. <https://tgeu.org/wp-content/uploads/2017/10/Overdiagnosed_Underserved-TransHealthSurvey.pdf> |
| **South Africa** | One outpatient department provides gender-affirming treatments:  D. Wilson, A. Marais, A. de Villiers, R. Addinall & M.M. Campbell (2014). Transgender issues in South Africa, with particular reference to the Groote Schuur Hospital Transgender Unit. SAMJ South African Medical Journal, 104(6).  Another gender clinic that provides gender-affirming treatments:  eNCA. (12. November 2019). SA gets first transgender healthcare facility. <https://www.enca.com/life/wits-opens-door-healthcare-trans-people>  Other local hospitals offer single treatments; access to transgender-specific treatments is difficult:  Zali, M. (31. März 2020). Transgender healthcare 'is a matter of human dignity'. Health-e News. <https://health-e.org.za/2020/03/31/transgender-healthcare-is-a-matter-of-human-dignity/> |
| **Spain** | Legal recognition after psychiatric diagnosis; some regions have gender clinics, some just local providers:  Smiley, A., Burgwal, A., Orre, C. & Summanen, E. (2017). Overdiagnosed but Underserved.: Trans Healthcare in Georgia, Poland, Serbia, Spain, and Sweden: Trans Health Survey. https://tgeu.org/wp-content/uploads/2017/10/Overdiagnosed_Underserved-TransHealthSurvey.pdf  One gender clinic is confirmed here (Methods section):  Gómez-Gil, E., Zubiaurre-Elorza, L., Antonio, I. E. de, Guillamon, A. & Salamero, M. (2014). Determinants of quality of life in Spanish transsexuals attending a gender unit before genital sex reassignment surgery. Quality of Life Research, 23(2), 669–676. <https://doi.org/10.1007/s11136-013-0497-3> |
| **Sweden** | List of gender identity teams or clinics:  <https://www.transformering.se/vardhalsa/transvard/utredningsteam>  One gender clinic is also named here:  Swedish Transgender Health \| Swedish Medical Center Seattle and Issaquah. (5. Oktober 2020). <https://www.swedish.org/services/transgender-health>  Six interdisciplinary gender teams or clinics are mentioned here:  ASHA Wire. (NaN). Interdisciplinary Program in Sweden Related to Transgender Voice. <https://pubs.asha.org/doi/abs/10.1044/vvd25.2.87> |
| **Switzerland** | Lack of experience in care providers:  Recher, A. (2017). On the Situation of Trans People in SWITZERLAND. https://tbinternet.ohchr.org/Treaties/CEDAW/Shared%20Documents/CHE/INT_CEDAW_NGO_CHE_23085_E.pdf  Two gender clinics; one interdisciplinary and publicly-funded; another one private and offering only gender-affirming surgeries:  Willkommen am UniversitätsSpital Zürich. (5. Oktober 2020). <http://www.plastische-chirurgie.usz.ch/fachwissen/PlastischeChirurgie/geschlechts-dysphorie/Seiten/default.aspx>  Zürich, O. C. (5. Oktober 2020). Transgender OP in Zürich \| OC Zürich. <https://www.oceanclinic.ch/transgender-op-zuerich/>  Available medical procedures in Switzerland:  TGNS Transgender Network Switzerland. (5. Oktober 2020). Medizin \| TGNS Transgender Network Switzerland. <https://www.tgns.ch/de/information/medizinisches/> |
| **Turkey** | One university hospital and other local clinics offer transgender-specific health care; lack of quality for the provided care:  Yılmaz, V. & Göçmen, İ. (2016). Denied Citizens of Turkey: Experiences of Discrimination Among LGBT Individuals in Employment, Housing and Health Care. Gender, Work & Organization, 23(5), 470–488. <https://doi.org/10.1111/gwao.12122> |
| **U.S.** | Liste with gender clinics and local providers offering gender-affirming treatments:  Transgender Care Listings \| The presence of a provider on this site does not constitute an endorsement or recommendation of that provider. Access care at your own risk. (5. Oktober 2020). <http://transcaresite.org/>  List of gender clinics offering gender-affirming treatments:  Trans Health. (5. Oktober 2020). Trans Health Clinics - Trans Health. <http://www.trans-health.com/clinics/> |
| **U.K.** | Eight NHS funded gender clinics offering gender-affirming treatments:  nhs.uk. (NaN). How to find an NHS gender dysphoria clinic. <https://www.nhs.uk/live-well/healthy-body/how-to-find-an-nhs-gender-identity-clinic/> |
| **Ukraine** | Gender-affirming treatments only offered by "sympathetic doctors ":  openDemocracy. (5. Oktober 2020). Being in Ukraine \| openDemocracy. <https://www.opendemocracy.net/en/odr/being-trans-in-ukraine/>  Access to transgender-specific health care is difficult because of legal barriers and the lack of experienced providers:  Ivantchenko, S. (2010). Situation of Transgender Persons in Ukraine. https://www.ilga-europe.org/sites/default/files/Attachments/ukraine_-_final_report_0.pdf  Legal recognition and barriers in Ukraine:  Human Rights Watch. (2017). A Glimmer of Light for Transgender People in Ukraine. <https://www.hrw.org/news/2017/01/27/glimmer-light-transgender-people-ukraine> |

Table S9 TransCareExpert survey - questionnaire

| **The Hamburg TransCareExpert Survey**  Dear Colleague,  Thank you for taking the time to participate in our expert survey.  The Hamburg TransCareExpert Survey is part of TransCare, a research project located at the Interdisciplinary Transgender Health Care Center, University Medical Center Hamburg, Germany. For further information, see [ResearchGate-link].  The TransCare study investigates whether and to what extent the setting for transgender health service delivery makes a difference in terms of psychosocial outcomes, such as distress. Centralized health care delivery settings are compared with decentralized delivery settings. A centralized delivery setting is one that offers all transition-related, gender-affirmative interventions at one location, e. g., a specialized gender clinic. In a decentralized delivery setting, different medical services carry out single treatments at various locations (Fig. 1).  In a first step, we conducted a systematic review and analyzed the present follow-up studies (period: 2000 to 2019) for possible effects of the health care delivery setting (central vs. decentralized, see above) on the outcome. For further information, see [Prospero-link]. To improve the weak evidence base on this matter, which stems from the review, we are now running this expert survey. Therefore, we are asking you as a clinician and/or researcher to answer a few questions regarding the setting in which you are providing transgender health care or health services research.  The survey findings are expected to improve our understanding of health care delivery structures with respect to the extent of centralization.  Participation involves completing the survey. The survey is anonymous and will take approx. XXX minutes to complete. We do not collect any data that enables us to identify you as a person. You will not have any direct personal benefits or harms from participating in this study. Participation is voluntary, and you can stop taking the survey at any time. The study received ethical approval from the local ethics committee of the University Medical Center Hamburg-Eppendorf, Germany (LPEK-0071)  If you have any questions, please contact *Andreas Koehler, MSc*, an.koehler@uke.de  Please take a moment to complete this survey. Thank you for your participation!  The Hamburg TransCare team  [BLINDED FOR REVIEW] | | | | | | |
| --- | --- | --- | --- | --- | --- | --- |
| **1.** | Do you provide gender-affirmative medical care (e.g., hormone therapy, surgery) to treat Gender Incongruence or Gender Dysphoria, or are you a researcher in the field of transgender health? [Multiple answer possible) | | | | | |
|  |  | |  | |  | |
|  | Yes | |  | |  | |
|  | | Medical care |  | |  | |
|  | | Mental health care |  | |  | |
|  | | Research |  | |  | |
|  | No | |  | |  | |
|  |  | |  | |  | |
| **3.** | What type of clinician are you? | | | | | |
|  |  | |  | |  | |
|  | Physician | |  | |  | |
|  | Advanced Practice Nurse | |  | |  | |
|  | Physician Assistant | |  | |  | |
|  | Another type of clinician | |  | |  | |
|  | Psychologist | |  | |  | |
|  | Licensed Clinical Social Worker | |  | |  | |
|  | Speech-Language Pathologist | |  | |  | |
|  |  | |  | |  | |
|  | What is your medical specialty? | |  | |  | |
|  |  | |  | |  | |
|  | surgery | |  | |  | |
|  | endocrinology | |  | |  | |
|  | mental health | |  | |  | |
|  | other, please specify: | |  | |  | |
|  |  | |  | |  | |
|  | Do you have any additional sub-specialty, fellowship training, or licensure comments? | | | | | |
|  |  | |  | |  | |
| **4.** | In which country do you mainly practice? | | | | | |
|  |  | |  | |  | |
| **5.** | How many years of experience do you have in working with transgender individuals? | | | | | |
|  |  | |  | |  | |
| **6.** | In what kind of institution do you work? | | | | | |
|  |  | |  | |  | |
|  | university hospital | |  | |  | |
|  | non-university hospital | |  | |  | |
|  | community health center | |  | |  | |
|  | private practice | |  | |  | |
|  | Other, please specify | |  | |  | |
|  |  | |  | |  | |
| **7.** | What kind of transgender-related treatment options does your institution offer? | | | | | |
|  |  | |  | |  | |
|  | mental health care | |  | |  | |
|  | hormonal treatment | |  | |  | |
|  | gender-affirmative surgery | |  | |  | |
|  | hair removal | |  | |  | |
|  | speech therapy | |  | |  | |
|  | others, please specify | |  | |  | |
|  |  | |  | |  | |
| **8.** | What is your research specialty? | |  | |  | |
|  |  | |  | |  | |
|  | Mental health | |  | |  | |
|  | Children & Adolescents | |  | |  | |
|  | Endocrinology | |  | |  | |
|  | Social Sciences | |  | |  | |
|  | Voice and Communication | |  | |  | |
|  | Surgery | |  | |  | |
|  | Law | |  | |  | |
|  | others, please specify | |  | |  | |
|  |  | |  | |  | |
| **9.** | How many years of experience do you have in research regarding transgender issues? | | | | | |
|  |  | |  | |  | |
| **10.** | In what kind of research institution do you work? | | | | | |
|  |  | |  | |  | |
|  | university/ university hospital | |  | |  | |
|  | public research institute (not related to a university) | |  | |  | |
|  | private research institute (not related to a university) | |  | |  | |
|  | others, please specify: | |  | |  | |
|  |  | |  | |  | |
| **11.** | What are the research foci at your institution in terms of transgender health care? | | | | | |
|  |  | |  | |  | |
|  | please describe: | |  | |  | |
|  |  | |  | |  | |
| **12.** | In some countries, transgender health care is primarily provided by a single institution, e.g., a specialized gender clinic. This kind of health care delivery setting is called **centralized**.  In other countries, transgender health care services are mostly offered by institutions from several locations. This kind of health care delivery setting is called **decentralized**. In some countries, both types of health care delivery settings are in use.  In which kind of health care delivery setting are you working?  [e.g., if you are a clinician at a specialized gender clinic offering most or all transition-related medical treatments, choose *centralized health care delivery setting*.] | | | | | |
|  |  | |  | |  | |
|  | centralized health care delivery setting | |  | |  | |
|  | decentralized health care delivery setting | |  | |  | |
|  |  | |  | |  | |
| **13.** | Considering the country in which you are working, in which kind of health care delivery setting is transgender health care primarily provided? | | | | | |
|  |  | |  | |  | |
|  | only centralized | |  | |  | |
|  | mostly centralized | |  | |  | |
|  | both centralized and decentralized | |  | |  | |
|  | mostly decentralized | |  | |  | |
|  | only decentralized | |  | |  | |
|  |  | |  | |  | |
| **14.** | Please describe the health care delivery setting of the country in which you are working in more detail. For example, see below. | | | | | |
|  |  | | | | | |
|  | [EXAMPLE: Transgender health care in the country of XYZ is provided in 5 public hospitals offering all necessary treatment options as well as by local health care professionals. Mostly, local health care professionals offer hormonal treatment and mental health care. There is a functioning network between health care professionals from gender clinics and local providers.] | | | | | |
|  |  | | | | | |
| **15.** | How does the health care delivery setting of the country in which you are working affect the involvement / active contribution of the transgender community in/to health care provision and research (e.g., establishing a community advisory board)? | | | | | |
|  |  | | | | | |
|  | positively, please describe | | | | | |
|  | negatively, please describe | | | | | |
|  | neither positively nor negatively, please describe | | | | | |
|  | I cannot judge/ I don’t know | | | | | |
|  |  | | | | | |
| **16.** | How does the health care delivery setting of the country in which you are working affect the professional exchange of expertise between institutions and providers of transgender health care (e.g., implementation of regular professional meetings)? | | | | | |
|  |  | | | | | |
|  | positively, please describe | | | | | |
|  | negatively, please describe | | | | | |
|  | neither positively nor negatively, please describe | | | | | |
|  | I cannot judge/ I don’t know | | | | | |
|  |  | | | | | |
| **17.** | How does the health care delivery setting of the country in which you are working affect the collaboration with non-medical community-linked institutions (e.g., advocacy groups, support groups)? | | | | | |
|  |  | | | | | |
|  | positively, please describe | | | | | |
|  | negatively, please describe | | | | | |
|  | neither positively nor negatively, please describe | | | | | |
|  | I cannot judge/ I don’t know | | | | | |
|  |  | | | | | |
| **17.a** | What are your personal experiences with the centralized and/or decentralized delivery of transgender health care? | | | | | |
|  | [FREE TEXT ANSWER] | | | | | |
|  |  | | | | | |
| **17.b** | What do you think are pros and cons for centralized and decentralized health care?  [TABLE WITH OPTION TO WRITE DOWN PROS AND CONS] | | | | | |
|  |  | | | | | |
| **17.c** | What do you think could be the effects of centralized and decentralized delivery of transgender health care services on transgender individuals seeking gender-affirming interventions?  [TABLE WITH OPTION TO WRITE DOWN POTENTIAL EFFECTS OF CENTRALIZED AND DECENTRALIZED DELIVERY] | | | | | |
|  |  | | | | | |
| **17.d** | What kind of health care delivery setting do you personally prefer? (independent from the setting you might be working in) | | | | | |
|  |  | | | | | |
|  | centralized health care delivery setting | | | | | |
|  | decentralized health care delivery setting | | | | | |
|  | I cannot judge/ I don’t know | | | | | |
|  |  | | | | | |
| **18.** | Please briefly describe the situation of health insurance coverage for gender-affirming interventions in the country in which you are working. | | | | | |
|  |  | | | | | |
| **18.** | How does the health care delivery setting of the country in which you are working affect the collaboration with health insurances? | | | | | |
|  |  | | | | | |
|  | positively, please describe | | | | | |
|  | negatively, please describe | | | | | |
|  | neither positively nor negatively, please describe | | | | | |
|  | I cannot judge/ I don’t know | | | | | |
|  |  | | | | | |
| **19.** | On average, how long does a transgender person seeking treatment have to wait for a first appointment in your country? | | | | | |
|  |  | | | | | |
|  | for mental health care: | | | | | |
|  | for hormone therapy: | | | | | |
|  | for surgery: | | | | | |
|  |  | | | | | |
| **19.** | Is there any additional comment or information you would like to add? | | | | | |
|  |  | | | | | |
|  | In the following, we would like you to answer a few short questions regarding demographics. This will help us to gain a more comprehensive understanding of your answers. | | | | | |
|  |  | | | | | |
| **20.** | How old are you? | | | | | |
|  |  | | | | | |
| **21.** | Please describe your gender identity: | | | | | |
|  |  | | |  | |  |
|  | Man/Male (men of transgender experience and cisgender men) | | |  | |  |
|  | Woman/Female (women of transgender experience and cisgender women) | | |  | |  |
|  | Non-binary/genderqueer | | |  | |  |
|  | other gender identity, please describe: | | |  | |  |
|  |  | | |  | |  |
| **22.** | Do you identify as a person of transgender experience? This includes different gender-diverse identities (e.g., non-binary and genderqueer). | | | | | |
|  |  | | |  | |  |
|  | Yes | | |  | |  |
|  | No | | |  | |  |
|  | I prefer not to answer this question | | |  | |  |
|  |  | | |  | |  |

### NIH Quality Assessment Tool for Before-After (Pre-Post) Studies With No Control Group

### Raters: [BLINDED FOR REVIEW]

| Ayden et al. (2015) | | | | |
| --- | --- | --- | --- | --- |
|  | **Criteria** | **Yes** | **No** | **Other (CD, NR, NA)*** |
| 1 | Was the study question or objective clearly stated? | x |  |  |
| 2 | Were eligibility/selection criteria for the study population prespecified and clearly described? | x |  |  |
| 3 | Were the participants in the study representative of those who would be eligible for the test/service/intervention in the general or clinical population of interest? | x |  |  |
| 4 | Were all eligible participants that met the prespecified entry criteria enrolled? |  | x |  |
| 5 | Was the sample size sufficiently large to provide confidence in the findings? |  |  | CD |
| 6 | Was the test/service/intervention clearly described and delivered consistently across the study population? |  | x |  |
| 7 | Were the outcome measures prespecified, clearly defined, valid, reliable, and assessed consistently across all study pa | x |  |  |
| 8 | Were the people assessing the outcomes blinded to the participants' exposures/interventions? |  | x |  |
| 9 | Was the loss to follow-up after baseline 20% or less? Were those lost to follow-up accounted for in the analysis? |  | x |  |
| 10 | Did the statistical methods examine changes in outcome measures from before to after the intervention? Were statistical tests done that provided p values for the pre-to-post changes? |  | x |  |
| 11 | Were outcome measures of interest taken multiple times before the intervention and multiple times after the intervention (i.e., did they use an interrupted time-series design)? |  | x |  |
| 12 | If the intervention was conducted at a group level (e.g., a whole hospital, a community, etc.) did the statistical analysis take into account the use of individual-level data to determine effects at the group level? |  |  | CD |

*CD, cannot determine; NA, not applicable; NR, not reported

| **Quality Rating** (**Good, Fair, or Poor) (see guidance)** | |
| --- | --- |
| Rater #1 Initials: | Fair |
| Rater #2 Initials: | Fair |
| Additional Comments (If POOR, please state why): |  |

| Costa et al. (2015) | | | | |
| --- | --- | --- | --- | --- |
|  | **Criteria** | **Yes** | **No** | **Other (CD, NR, NA)*** |
| 1 | Was the study question or objective clearly stated? | x |  |  |
| 2 | Were eligibility/selection criteria for the study population prespecified and clearly described? | x |  |  |
| 3 | Were the participants in the study representative of those who would be eligible for the test/service/intervention in the general or clinical population of interest? |  |  | CD |
| 4 | Were all eligible participants that met the prespecified entry criteria enrolled? | x |  |  |
| 5 | Was the sample size sufficiently large to provide confidence in the findings? | x |  |  |
| 6 | Was the test/service/intervention clearly described and delivered consistently across the study population? | x |  |  |
| 7 | Were the outcome measures prespecified, clearly defined, valid, reliable, and assessed consistently across all study pa | x |  |  |
| 8 | Were the people assessing the outcomes blinded to the participants' exposures/interventions? |  | x |  |
| 9 | Was the loss to follow-up after baseline 20% or less? Were those lost to follow-up accounted for in the analysis? |  | x |  |
| 10 | Did the statistical methods examine changes in outcome measures from before to after the intervention? Were statistical tests done that provided p values for the pre-to-post changes? | x |  |  |
| 11 | Were outcome measures of interest taken multiple times before the intervention and multiple times after the intervention (i.e., did they use an interrupted time-series design)? |  | x |  |
| 12 | If the intervention was conducted at a group level (e.g., a whole hospital, a community, etc.) did the statistical analysis take into account the use of individual-level data to determine effects at the group level? |  |  | NA |

*CD, cannot determine; NA, not applicable; NR, not reported

| **Quality Rating** (**Good, Fair, or Poor) (see guidance)** | |
| --- | --- |
| Rater #1 Initials: | Fair |
| Rater #2 Initials: | Fair |
| Additional Comments (If POOR, please state why): |  |

| De Cuypere et al. (2005) | | | | |
| --- | --- | --- | --- | --- |
|  | **Criteria** | **Yes** | **No** | **Other (CD, NR, NA)*** |
| 1 | Was the study question or objective clearly stated? | x |  |  |
| 2 | Were eligibility/selection criteria for the study population prespecified and clearly described? | x |  |  |
| 3 | Were the participants in the study representative of those who would be eligible for the test/service/intervention in the general or clinical population of interest? |  |  | CD |
| 4 | Were all eligible participants that met the prespecified entry criteria enrolled? |  | x |  |
| 5 | Was the sample size sufficiently large to provide confidence in the findings? | x |  |  |
| 6 | Was the test/service/intervention clearly described and delivered consistently across the study population? | x |  |  |
| 7 | Were the outcome measures prespecified, clearly defined, valid, reliable, and assessed consistently across all study pa | x |  |  |
| 8 | Were the people assessing the outcomes blinded to the participants' exposures/interventions? | x |  |  |
| 9 | Was the loss to follow-up after baseline 20% or less? Were those lost to follow-up accounted for in the analysis? |  | x |  |
| 10 | Did the statistical methods examine changes in outcome measures from before to after the intervention? Were statistical tests done that provided p values for the pre-to-post changes? | x |  |  |
| 11 | Were outcome measures of interest taken multiple times before the intervention and multiple times after the intervention (i.e., did they use an interrupted time-series design)? |  | x |  |
| 12 | If the intervention was conducted at a group level (e.g., a whole hospital, a community, etc.) did the statistical analysis take into account the use of individual-level data to determine effects at the group level? |  | x |  |

*CD, cannot determine; NA, not applicable; NR, not reported

| **Quality Rating** (**Good, Fair, or Poor) (see guidance)** | |
| --- | --- |
| Rater #1 Initials: | poor |
| Rater #2 Initials: | poor |
| Additional Comments (If POOR, please state why): | based on knowledge by cooperation, we know about the situation of trans health care in BEL. Comparable to NL, but separated between the Dutch and the French speaking part |

| Deutsch et al. (2015) | | | | |
| --- | --- | --- | --- | --- |
|  | **Criteria** | **Yes** | **No** | **Other (CD, NR, NA)*** |
| 1 | Was the study question or objective clearly stated? | x |  |  |
| 2 | Were eligibility/selection criteria for the study population prespecified and clearly described? | x |  |  |
| 3 | Were the participants in the study representative of those who would be eligible for the test/service/intervention in the general or clinical population of interest? |  |  | CD |
| 4 | Were all eligible participants that met the prespecified entry criteria enrolled? |  |  | CD |
| 5 | Was the sample size sufficiently large to provide confidence in the findings? |  | x |  |
| 6 | Was the test/service/intervention clearly described and delivered consistently across the study population? | x |  |  |
| 7 | Were the outcome measures prespecified, clearly defined, valid, reliable, and assessed consistently across all study pa |  | x |  |
| 8 | Were the people assessing the outcomes blinded to the participants' exposures/interventions? |  | x |  |
| 9 | Was the loss to follow-up after baseline 20% or less? Were those lost to follow-up accounted for in the analysis? |  |  | CD |
| 10 | Did the statistical methods examine changes in outcome measures from before to after the intervention? Were statistical tests done that provided p values for the pre-to-post changes? |  | x |  |
| 11 | Were outcome measures of interest taken multiple times before the intervention and multiple times after the intervention (i.e., did they use an interrupted time-series design)? |  | x |  |
| 12 | If the intervention was conducted at a group level (e.g., a whole hospital, a community, etc.) did the statistical analysis take into account the use of individual-level data to determine effects at the group level? |  | x |  |

*CD, cannot determine; NA, not applicable; NR, not reported

| **Quality Rating** (**Good, Fair, or Poor) (see guidance)** | |
| --- | --- |
| Rater #1 Initials: | poor |
| Rater #2 Initials: | poor |
| Additional Comments (If POOR, please state why): | - |

| Dhejne et al. (2011) | | | | |
| --- | --- | --- | --- | --- |
|  | **Criteria** | **Yes** | **No** | **Other (CD, NR, NA)*** |
| 1 | Was the study question or objective clearly stated? | x |  |  |
| 2 | Were eligibility/selection criteria for the study population prespecified and clearly described? | x |  |  |
| 3 | Were the participants in the study representative of those who would be eligible for the test/service/intervention in the general or clinical population of interest? |  |  | CD |
| 4 | Were all eligible participants that met the prespecified entry criteria enrolled? |  | x |  |
| 5 | Was the sample size sufficiently large to provide confidence in the findings? | x |  |  |
| 6 | Was the test/service/intervention clearly described and delivered consistently across the study population? | x |  |  |
| 7 | Were the outcome measures prespecified, clearly defined, valid, reliable, and assessed consistently across all study pa | x |  |  |
| 8 | Were the people assessing the outcomes blinded to the participants' exposures/interventions? |  | x |  |
| 9 | Was the loss to follow-up after baseline 20% or less? Were those lost to follow-up accounted for in the analysis? |  | x |  |
| 10 | Did the statistical methods examine changes in outcome measures from before to after the intervention? Were statistical tests done that provided p values for the pre-to-post changes? | x |  |  |
| 11 | Were outcome measures of interest taken multiple times before the intervention and multiple times after the intervention (i.e., did they use an interrupted time-series design)? |  | x |  |
| 12 | If the intervention was conducted at a group level (e.g., a whole hospital, a community, etc.) did the statistical analysis take into account the use of individual-level data to determine effects at the group level? |  | x |  |

*CD, cannot determine; NA, not applicable; NR, not reported

| **Quality Rating** (**Good, Fair, or Poor) (see guidance)** | |
| --- | --- |
| Rater #1 Initials: | poor |
| Rater #2 Initials: | poor |
| Additional Comments (If POOR, please state why): | no info on how patients were referred |

| Gooren et al. (2013) | | | | |
| --- | --- | --- | --- | --- |
|  | **Criteria** | **Yes** | **No** | **Other (CD, NR, NA)*** |
| 1 | Was the study question or objective clearly stated? | x |  |  |
| 2 | Were eligibility/selection criteria for the study population prespecified and clearly described? | x |  |  |
| 3 | Were the participants in the study representative of those who would be eligible for the test/service/intervention in the general or clinical population of interest? |  |  | CD |
| 4 | Were all eligible participants that met the prespecified entry criteria enrolled? |  | x |  |
| 5 | Was the sample size sufficiently large to provide confidence in the findings? | x |  |  |
| 6 | Was the test/service/intervention clearly described and delivered consistently across the study population? |  | x |  |
| 7 | Were the outcome measures prespecified, clearly defined, valid, reliable, and assessed consistently across all study pa |  | x |  |
| 8 | Were the people assessing the outcomes blinded to the participants' exposures/interventions? |  | x |  |
| 9 | Was the loss to follow-up after baseline 20% or less? Were those lost to follow-up accounted for in the analysis? |  | x |  |
| 10 | Did the statistical methods examine changes in outcome measures from before to after the intervention? Were statistical tests done that provided p values for the pre-to-post changes? |  |  | CD |
| 11 | Were outcome measures of interest taken multiple times before the intervention and multiple times after the intervention (i.e., did they use an interrupted time-series design)? |  | c |  |
| 12 | If the intervention was conducted at a group level (e.g., a whole hospital, a community, etc.) did the statistical analysis take into account the use of individual-level data to determine effects at the group level? |  |  | CD |

*CD, cannot determine; NA, not applicable; NR, not reported

| **Quality Rating** (**Good, Fair, or Poor) (see guidance)** | |
| --- | --- |
| Rater #1 Initials: | fair |
| Rater #2 Initials: | fair |
| Additional Comments (If POOR, please state why): | but only because we know about the transgender health care situation in NL based on cooperation |

| Heylens et al. (2014) | | | | |
| --- | --- | --- | --- | --- |
|  | **Criteria** | **Yes** | **No** | **Other (CD, NR, NA)*** |
| 1 | Was the study question or objective clearly stated? | x |  |  |
| 2 | Were eligibility/selection criteria for the study population prespecified and clearly described? | x |  |  |
| 3 | Were the participants in the study representative of those who would be eligible for the test/service/intervention in the general or clinical population of interest? |  |  | CD |
| 4 | Were all eligible participants that met the prespecified entry criteria enrolled? |  | x |  |
| 5 | Was the sample size sufficiently large to provide confidence in the findings? | x |  |  |
| 6 | Was the test/service/intervention clearly described and delivered consistently across the study population? |  | x |  |
| 7 | Were the outcome measures prespecified, clearly defined, valid, reliable, and assessed consistently across all study pa | x |  |  |
| 8 | Were the people assessing the outcomes blinded to the participants' exposures/interventions? |  | x |  |
| 9 | Was the loss to follow-up after baseline 20% or less? Were those lost to follow-up accounted for in the analysis? | x |  |  |
| 10 | Did the statistical methods examine changes in outcome measures from before to after the intervention? Were statistical tests done that provided p values for the pre-to-post changes? | x |  |  |
| 11 | Were outcome measures of interest taken multiple times before the intervention and multiple times after the intervention (i.e., did they use an interrupted time-series design)? |  | x |  |
| 12 | If the intervention was conducted at a group level (e.g., a whole hospital, a community, etc.) did the statistical analysis take into account the use of individual-level data to determine effects at the group level? |  | x |  |

*CD, cannot determine; NA, not applicable; NR, not reported

| **Quality Rating** (**Good, Fair, or Poor) (see guidance)** | |
| --- | --- |
| Rater #1 Initials: | poor |
| Rater #2 Initials: | poor |
| Additional Comments (If POOR, please state why): | - |

| Johannson et al. (2010) | | | | |
| --- | --- | --- | --- | --- |
|  | **Criteria** | **Yes** | **No** | **Other (CD, NR, NA)*** |
| 1 | Was the study question or objective clearly stated? | x |  |  |
| 2 | Were eligibility/selection criteria for the study population prespecified and clearly described? |  | x |  |
| 3 | Were the participants in the study representative of those who would be eligible for the test/service/intervention in the general or clinical population of interest? |  |  | CD |
| 4 | Were all eligible participants that met the prespecified entry criteria enrolled? |  | x |  |
| 5 | Was the sample size sufficiently large to provide confidence in the findings? |  | x |  |
| 6 | Was the test/service/intervention clearly described and delivered consistently across the study population? |  | x |  |
| 7 | Were the outcome measures prespecified, clearly defined, valid, reliable, and assessed consistently across all study pa |  | x |  |
| 8 | Were the people assessing the outcomes blinded to the participants' exposures/interventions? |  | x |  |
| 9 | Was the loss to follow-up after baseline 20% or less? Were those lost to follow-up accounted for in the analysis? |  | x |  |
| 10 | Did the statistical methods examine changes in outcome measures from before to after the intervention? Were statistical tests done that provided p values for the pre-to-post changes? | x |  |  |
| 11 | Were outcome measures of interest taken multiple times before the intervention and multiple times after the intervention (i.e., did they use an interrupted time-series design)? |  | x |  |
| 12 | If the intervention was conducted at a group level (e.g., a whole hospital, a community, etc.) did the statistical analysis take into account the use of individual-level data to determine effects at the group level? |  | x |  |

*CD, cannot determine; NA, not applicable; NR, not reported

| **Quality Rating** (**Good, Fair, or Poor) (see guidance)** | |
| --- | --- |
| Rater #1 Initials: | poor |
| Rater #2 Initials: | poor |
| Additional Comments (If POOR, please state why): | - |

| Simonsen et al. (2016) | | | | |
| --- | --- | --- | --- | --- |
|  | **Criteria** | **Yes** | **No** | **Other (CD, NR, NA)*** |
| 1 | Was the study question or objective clearly stated? | x |  |  |
| 2 | Were eligibility/selection criteria for the study population prespecified and clearly described? | x |  |  |
| 3 | Were the participants in the study representative of those who would be eligible for the test/service/intervention in the general or clinical population of interest? |  |  | CD |
| 4 | Were all eligible participants that met the prespecified entry criteria enrolled? |  | x |  |
| 5 | Was the sample size sufficiently large to provide confidence in the findings? | x |  |  |
| 6 | Was the test/service/intervention clearly described and delivered consistently across the study population? |  | x |  |
| 7 | Were the outcome measures prespecified, clearly defined, valid, reliable, and assessed consistently across all study pa |  | x |  |
| 8 | Were the people assessing the outcomes blinded to the participants' exposures/interventions? |  | x |  |
| 9 | Was the loss to follow-up after baseline 20% or less? Were those lost to follow-up accounted for in the analysis? |  |  | CD |
| 10 | Did the statistical methods examine changes in outcome measures from before to after the intervention? Were statistical tests done that provided p values for the pre-to-post changes? | x |  |  |
| 11 | Were outcome measures of interest taken multiple times before the intervention and multiple times after the intervention (i.e., did they use an interrupted time-series design)? |  | x |  |
| 12 | If the intervention was conducted at a group level (e.g., a whole hospital, a community, etc.) did the statistical analysis take into account the use of individual-level data to determine effects at the group level? |  |  |  |

*CD, cannot determine; NA, not applicable; NR, not reported

| **Quality Rating** (**Good, Fair, or Poor) (see guidance)** | |
| --- | --- |
| Rater #1 Initials: | fair |
| Rater #2 Initials: |  |
| Additional Comments (If POOR, please state why): |  |

| Simonsen et al. (2016) | | | | |
| --- | --- | --- | --- | --- |
|  | **Criteria** | **Yes** | **No** | **Other (CD, NR, NA)*** |
| 1 | Was the study question or objective clearly stated? | x |  |  |
| 2 | Were eligibility/selection criteria for the study population prespecified and clearly described? | x |  |  |
| 3 | Were the participants in the study representative of those who would be eligible for the test/service/intervention in the general or clinical population of interest? |  |  | CD |
| 4 | Were all eligible participants that met the prespecified entry criteria enrolled? |  | x |  |
| 5 | Was the sample size sufficiently large to provide confidence in the findings? | x |  |  |
| 6 | Was the test/service/intervention clearly described and delivered consistently across the study population? |  | x |  |
| 7 | Were the outcome measures prespecified, clearly defined, valid, reliable, and assessed consistently across all study pa |  | x |  |
| 8 | Were the people assessing the outcomes blinded to the participants' exposures/interventions? |  | x |  |
| 9 | Was the loss to follow-up after baseline 20% or less? Were those lost to follow-up accounted for in the analysis? |  |  | CD |
| 10 | Did the statistical methods examine changes in outcome measures from before to after the intervention? Were statistical tests done that provided p values for the pre-to-post changes? | x |  |  |
| 11 | Were outcome measures of interest taken multiple times before the intervention and multiple times after the intervention (i.e., did they use an interrupted time-series design)? |  | x |  |
| 12 | If the intervention was conducted at a group level (e.g., a whole hospital, a community, etc.) did the statistical analysis take into account the use of individual-level data to determine effects at the group level? |  |  |  |

*CD, cannot determine; NA, not applicable; NR, not reported

| **Quality Rating** (**Good, Fair, or Poor) (see guidance)** | |
| --- | --- |
| Rater #1 Initials: | poor |
| Rater #2 Initials: | poor |
| Additional Comments (If POOR, please state why): | no further info provided compared to the other Simonsen paper |

| **Wiepjes et al. (2019)** | | | | |
| --- | --- | --- | --- | --- |
|  | **Criteria** | **Yes** | **No** | **Other (CD, NR, NA)*** |
| 1 | Was the study question or objective clearly stated? | x |  |  |
| 2 | Were eligibility/selection criteria for the study population prespecified and clearly described? |  | x |  |
| 3 | Were the participants in the study representative of those who would be eligible for the test/service/intervention in the general or clinical population of interest? |  |  | CD |
| 4 | Were all eligible participants that met the prespecified entry criteria enrolled? |  | x |  |
| 5 | Was the sample size sufficiently large to provide confidence in the findings? | x |  |  |
| 6 | Was the test/service/intervention clearly described and delivered consistently across the study population? |  | x |  |
| 7 | Were the outcome measures prespecified, clearly defined, valid, reliable, and assessed consistently across all study pa | x |  |  |
| 8 | Were the people assessing the outcomes blinded to the participants' exposures/interventions? |  | x |  |
| 9 | Was the loss to follow-up after baseline 20% or less? Were those lost to follow-up accounted for in the analysis? |  | x |  |
| 10 | Did the statistical methods examine changes in outcome measures from before to after the intervention? Were statistical tests done that provided p values for the pre-to-post changes? | x |  |  |
| 11 | Were outcome measures of interest taken multiple times before the intervention and multiple times after the intervention (i.e., did they use an interrupted time-series design)? |  | x |  |
| 12 | If the intervention was conducted at a group level (e.g., a whole hospital, a community, etc.) did the statistical analysis take into account the use of individual-level data to determine effects at the group level? |  |  |  |

*CD, cannot determine; NA, not applicable; NR, not reported

| **Quality Rating** (**Good, Fair, or Poor) (see guidance)** | |
| --- | --- |
| Rater #1 Initials: | poor |
| Rater #2 Initials: | poor |
| Additional Comments (If POOR, please state why): | - |
